# Prenylated chromones and flavonoids isolated from the roots of *Flemingia macrophylla* and their anti-lung cancer activity

**DOI:** 10.1186/s13020-023-00860-3

**Published:** 2023-11-23

**Authors:** Baolin Wang, Qinqin Wang, Renyikun Yuan, Shilin Yang, Meilin Lu, Fuhong Yuan, Zhidan Dong, Menghuan Mo, Qiming Pan, Hongwei Gao

**Affiliations:** 1 College of Pharmacy, Guangxi University of Chinese Medicine, Nanning, 530200 China; 2 College of Pharmacy, Jiangxi University of Traditional Chinese Medicine, Nanchang, 330004 China; 3 Guangxi Engineering Technology Research Center of Advantage Chinese Patent Drug and Ethnic Drug Development, Nanning, 530020 China

## Abstract

**Background:**

The successful launch of icaritin, a therapeutic drug for liver cancer derived from *Epimedium brevicornu*, has provided new impetus for the development of prenylated flavonoids in the field of oncology.* Flemingia macrophyll*a is reported to contain characteristic prenylated flavonoids which can regulate the p53 protein. We aimed to isolate these constituents and conduct activity evaluation, structure–activity relationship, and mechanism studies to provide candidate compounds for antitumor drug development.

**Methods:**

In this study, chromatographic techniques combined with spectroscopic methods were used to separate, purify, and identify the constituents of *Flemingia macrophylla* methanol extract. The cytotoxic activity of the constituents was evaluated using an MTT assay with A549 and H1975 cells as the model. The binding mechanism between the compounds and the p53 protein was investigated with molecular docking and validated with cellular thermal shift assay (CETSA). Western blotting (WB) was employed to detect the expression of p53 protein and apoptosis-related proteins in cells.

**Results:**

Chiral HPLC separation of racemates **1** and **7** provided two pairs of undescribed enantiomers (**1a**/**1b** and **7a**/**7b**), along with eight known compounds (**2** − **9**) isolated from *Flemingia macrophylla* roots. Their structures were elucidated by spectroscopic analysis, and the absolute configurations of the enantiomers were determined from experimental and calculated electronic circular dichroism data. Compounds **1** − **7**, and the non-prenyl analogues **10** − **13**, were evaluated for cytotoxic activity against the human lung cancer A549 and H1975 cell line. Compounds 5 − 7 displayed better cytotoxicity than the positive control icaritin in A549 and H1975, with IC_50_ values ranging from 4.50 to 19.83 μmol·L^-1^ and < 5 μmol·L^-1^, respectively. The structure–activity relationships of the chromone or flavonoid analogues against A549 cells were discussed. Molecular docking results demonstrated that compound **7a** has strong interaction with p53 and WB indicated that **7a** induced apoptosis by increasing the p53 protein, decreasing the anti-apoptotic protein Bcl-2, and activating the caspase family in A549 cells. These results suggest that prenylated flavonoids are potential p53 protein activators.

**Conclusion:**

This study demonstrates that *Flemingia macrophylla* is rich in prenylated flavonoid constituents, among which compounds 5 and 7 exhibited significant cytotoxic activity against A549 cells and served as reference candidates for the design and development of prenylated compounds as antitumor therapeutic drugs.

**Graphical Abstract:**

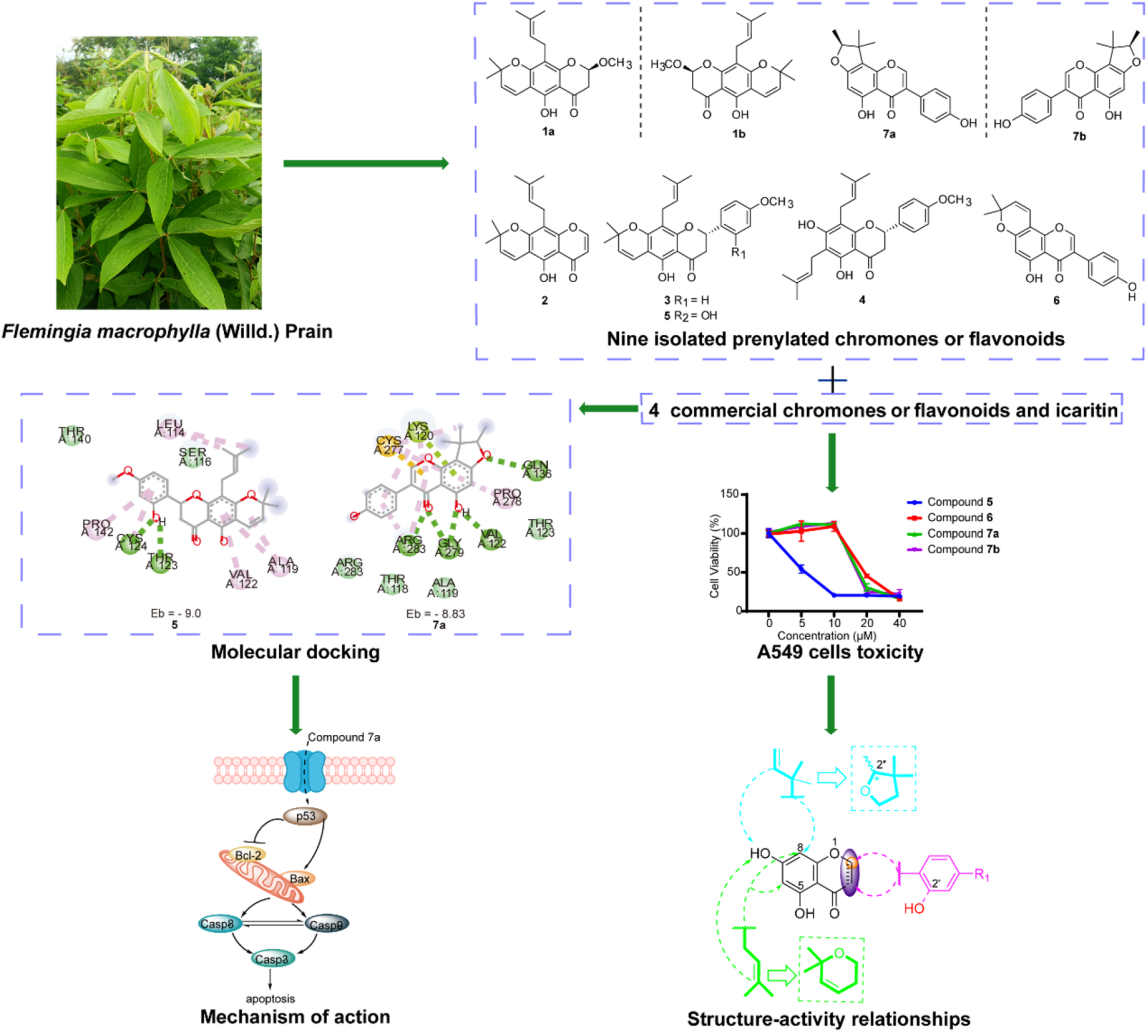

**Supplementary Information:**

The online version contains supplementary material available at 10.1186/s13020-023-00860-3.

## Highlights

- Two pairs of undescribed enantiomers (**1a/1b** and **7a/7b**) were isolated from *Flemingia macrophylla*.
- Prenylated flavonoids **5** − **7** exhibited significant cytotoxicity against A549 cells.
- Structure-activity relationship study revealed that the presence of B-ring and 2ʹ-OH substitution and the cyclization of 6/8-pentenyl group with 7-OH were beneficial for cytotoxicity.
- Molecular docking results showed that Cys124 and Cys277 were the key amino acid residues for binding.
- Compound **7a** induced apoptosis of A549 cells by activating the p53 protein.

**Supplementary Information:**

The online version contains supplementary material available at 10.1186/s13020-023-00860-3.

## Introduction

According to the Global Cancer Observatory (GLOBOCAN) in 2020, lung cancer is the second most diagnosed cancer and is the most prevalent cause of cancer deaths [1]. Non-small cell lung cancer (NSCLC) accounts for 85% of lung cancer cases [2]. Despite recent advancements in lung cancer treatment, the overall survival rate of advanced lung cancer patients is unsatisfactory. Therefore, there is an urgent need for effective medications for NSCLC with lower toxicity and less drug resistance. The p53 tumor suppressor protein acts as a major barrier against cancer initiation and progression [3]. It can inhibit the proliferation and promote apoptosis of lung cancer cells [4], so exploring drugs that restore the function of wild-type p53 is of great significance. Recently, icaritin, a natural prenylated flavonoid, has garnered attention as a drug for treating advanced liver cancer and for its inhibition of various solid tumors including lung, breast and esophageal cancers [5–7]. Icaritin induces cancer cell apoptosis and inhibits tumor growth through activation of p53 and suppression of Akt/mTOR pathways [8].

*Flemingia macrophylla* (Willd.) Merr., a shrub belonging to the Fabaceae family, is widely distributed in southern China, India and Indonesia [9, 10]. *F. macrophylla* is a traditional Zhuang and Yao medicine in China, and its nutritional value is well known. The roots have been used to dispel wind and dampness, strengthen bones and muscles, and promote blood circulation and detoxification [11]. There are more than 37 Chinese medicine preparations comprising *F. macrophylla*, including Fuke Qianjin, Jinji and Zhuangyao Jianshen formulations. Previous chemical investigations have demonstrated that *F. macrophylla* mainly contains flavones, isoflavones, flavonoid glycosides, chalcones, terpenoids, and phenolics [12–16], among which prenylated flavonoids are the major bioactive constituents, exhibiting anti-cancer, antioxidant, neuroprotective and analgesic activities [17–19].

**Fig. 1:**
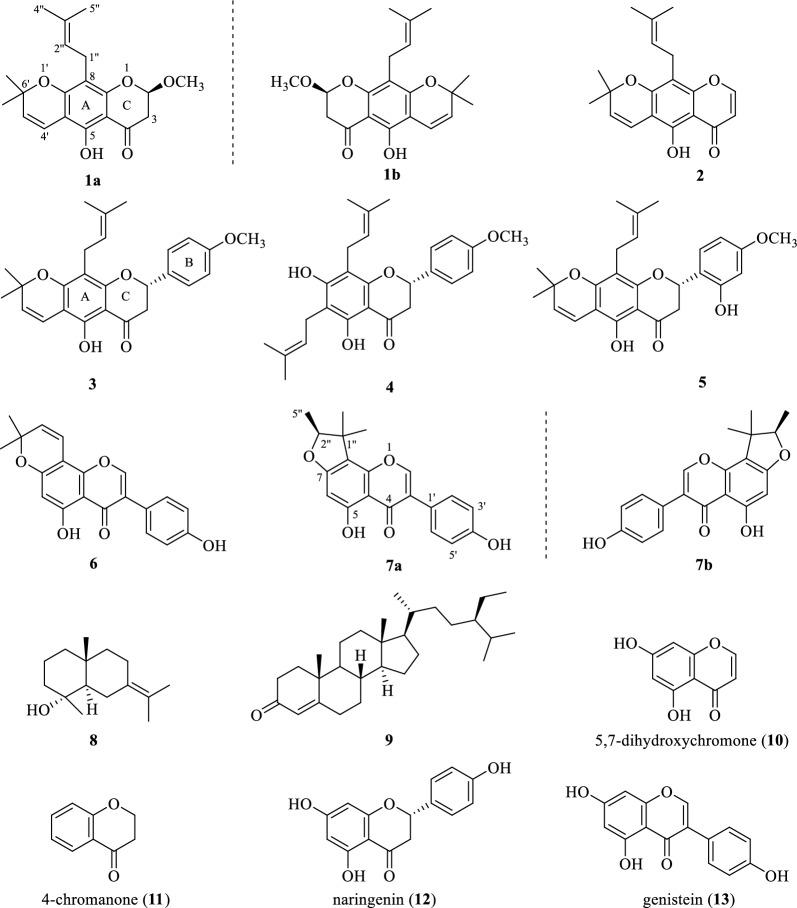
Structures of compounds** 1** − **9** isolated from *F. macrophylla* and the purchased non-prenyl analogues **10** − **13**

Therefore, the constituents of *F. macrophylla* root MeOH extract were investigated in search of novel prenylated flavonoids with cytotoxic activity for cancer therapy or prevention. As a result, two pairs of new chiral isomers and seven known compounds **2** − **9** were isolated. Chiral HPLC separation of racemic **1** and **7** provided two pairs of undescribed enantiomers (**1a**/**1b** and **7a**/**7b**, Fig. 1) whose absolute configurations were determined on the basis of experimental and calculated electronic circular dichroism (ECD). The cytotoxicity of **1** − **7** against A549 and H1975 cells was evaluated using the MTT method. To analyze the structure–activity relationship (SAR), commercial non-prenyl analogues **10** − **13** were also evaluated for cytotoxic activity against A549 cells. Molecular docking was used to predict the targets and binding modes of prenylated chromones and flavonoids to p53, and the results were confirmed by cellular thermal shift assay (CETSA). Western blot analysis of apoptosis-related proteins showed that compound **7a** can induce A549 cell apoptosis via a pro-apoptotic mechanism.

## Experimental

### General experimental procedures

IR spectra (KBr pellet) were recorded on a Nicolet iS10 spectrometer (Thermo Fisher Scientific Inc., Waltham, MA, USA). UV spectra were acquired with a METASH UV-5200 spectrophotometer (Yuanxi Instrument Co., Ltd, Shanghai, China). Optical rotations were obtained on a WZZ-3 automatic polarimeter (Yidian Wuguang, Shanghai, China). HR-ESI–MS was performed on a Waters ACQUITY UPLC/Xevo G2-XS Q-Tof mass spectrometer (Waters Corp., Milford, MA, USA). NMR spectra were obtained on a Bruker Avance III 500 MHz NMR instrument (Bruker Corporation, Switzerland). Semi-preparative HPLC separations were performed on a Shimadzu LC-20AD XR instrument equipped with an SPD-20A detector using a Waters SunFire C18 OBD column (10 mm × 250 mm, 5 µm) or a COSMOSIL Cholester column (10 mm × 250 mm, 5 µm). Chiral separation was performed using a Hplcone CHiRALONE 5-C column (10 mm × 250 mm, 5 µm, Suzhou Suyan Medical Technology Co., Ltd, Suzhou, China). Sephadex LH-20 (Cytiva, USA), silica gel (100 − 200, 200 − 300 mesh, Qingdao Marine Chemical Factory, Qingdao, China) and ODS (5 μm, Daisogel, Japan) were applied for column chromatography (CC). TLC was carried out with GF254 plates (Qingdao Marine Chemical Factory, Qingdao, China). Methanol and acetonitrile for HPLC were purchased from Thermo Fisher Scientific (Waltham, MA, USA). Deuterated solvents for NMR experiments were purchased from Sigma-Aldrich Chemical Co. (St. Louis, MO, USA). All analytical reagents were obtained from Kelong Chemical Co., Ltd (Chengdu, China). A549 and H1975 cells were purchased from ATCC. RPMI 1640, trypsin, and FBS were purchased from Life Technologies/Gibco Laboratories (Grand Island, NY, USA). MTT was purchased from Sigma-Aldrich (St. Louis, MO, USA). Antibodies for GAPDH (#8884), p53 (#E2422), pro-Casp3/8/9 (9694S, 7273 T) and cleaved-Casp3/8/9 (AF7022), and secondary antibodies including horseradish peroxidase (HRP)-conjugated goat anti-rabbit IgG (#7074) were purchased from Cell Signaling Technology (CST, Danvers, MA, USA). BCA protein assay kit, PVDF membranes, and Dual-Glo luciferase assay system kit were bought from Thermo Fisher (Waltham, MA, USA). Genistein (CR ≥ 98.0%, PS000784) was purchased from Push Bio-Technology Co. Ltd (Chengdu, China). Icaritin (HPLC ≥ 98.0%, C14875408) was purchased from Macklin Biochemical co., Ltd (Shanghai, China), 4-chromanone (98.0%, LP10S57) was purchased from J&K Scientific (Beijing, China), 5,7-dihydroxychromone (98%, J2213702) was purchased from Aladdin Bio-Technology Co., Ltd, and naringenin (HPLC ≥ 98.40%, MUST-22072711) was purchased from MUST Bio-Technology Co., Ltd (Chengdu, China).

### Plant material

*F. macrophylla* roots were purchased from Nanbei Hang Traditional Chinese Medicine Decoction Pieces Co. Ltd, Guangzhou, in October 2020, and were identified by Professor Liu-Ping Wang of Guangxi University of Chinese Medicine. A voucher specimen (No. 20201002) was deposited at the Guangxi Scientific Research Center of Traditional Chinese Medicine, Guangxi University of Chinese Medicine.

### Extraction and isolation

The dried and powdered roots of *F. macrophylla* (92.7 kg) were extracted five times with methanol (40 L) at room temperature for 3 days. After concentration *in vacuo*, the extract (29.7 kg) was suspended in water, and was partitioned successively with dichloromethane, EtOAc and *n*-butanol (3 extractions per solvent), yielding the dichloromethane fraction (2.5 kg), EtOAc fraction (1.0 kg) and *n*-butanol fraction (1.0 kg). The dichloromethane fraction (2.5 kg) was subjected to silica gel CC eluting with a gradient of cyclohexane-EtOAc (40:1 to 0:1, v/v) to afford fractions A − J.

Fraction D (280.0 g) was further separated by reversed-phase C_18_ (RP-C_18_) silica gel CC eluting with MeOH-H_2_O (70:30 to 100:0) to provide 27 subfractions (D1 − D27). D11 (0.25 g) was further purified by semi-preparative HPLC (MeOH-H_2_O, 85:15 v/v, 2 ml/min) to afford compound **8** (62.5 mg, *t*_R_ 15.3 min). D12 (1.8 g) was separated on a Sephadex LH-20 column (CH_2_Cl_2_-MeOH, 1:1) to afford D12.1 − D12.4. Fraction D12.2 (0.34 g) was purified by semi-preparative HPLC (COSMOSIL Cholester column, MeOH-H_2_O-TFA, 90:10:0.1, 3 ml/min) to give compound **4** (22.3 mg, *t*_R_ 28.2 min). D13 (2.3 g) was separated by silica gel CC (cyclohexane-EtOAc, 80:1 to 20:1) to provide 5 subfractions (D13.1 − D13.5). D13.3 (0.21 g) was purified by semi-preparative HPLC (COSMOSIL Cholester column, MeOH-H_2_O-TFA, 90:10:0.1, 3 ml/min) to yield compounds **1** (29.5 mg, *t*_R_ 24.6 min) and **2** (28.3 mg, *t*_R_ 25.9 min). The enantiomers of compound 1 were separated by chiral HPLC using a Hplcone CHiRALONE 5-C column (*n*-hexane–isopropanol, 90:10, 2 ml/min) to afford **1a** (2.3 mg, *t*_R_ 16.0 min) and **1b** (2.4 mg, *t*_R_ 17.2 min). D16 (0.35 g) was fractioned using semi-preparative HPLC (MeOH-H_2_O, 84:16) to obtain D16.1 − D16.4. Fraction D16.3 (0.19 g) was purified on a Sephadex LH-20 column (CH_2_Cl_2_-MeOH, 1:1) to give compound **3** (21.3 mg). D27 (0.53 g) was purified by semi-preparative HPLC (MeOH-H_2_O, 100:0, 2 ml/min) to afford compound **9** (56.4 mg, *t*_R_ 15.3 min).

Fraction F (627.5 g) was applied to silica gel CC (cyclohexane-EtOAc, 100:1 to 40:1) to provide F1 − F5. Fraction F3 (0.50 g) was separated on a Sephadex LH-20 column (CH_2_Cl_2_-MeOH, 1:1) to give compound **5** (128.4 mg). Fraction H (21.5 g) was separated by RP-C_18_ silica gel CC (MeOH-H_2_O, 60:30 to 100:0) to provide 8 subfractions (H1-H8). Fraction H2 (0.16 g) was purified by semi-preparative HPLC (CH_3_CN-H_2_O, 60:40, 2 mL/min) to give compounds **6** (15.4 mg, *t*_R_ 18.5 min) and **7** (42.5 mg, *t*_R_ 29.3 min). Compound **7** was resolved by chiral-phase HPLC on a Hplcone CHiRALONE 5-C column (*n*-hexane–isopropanol, 90:10, 2 mL/min) to afford **7a** (8.3 mg, *t*_R_ 12.8 min) and **7b** (9.6 mg, *t*_R_ 16.7 min).

2-*O*-Methyl eriosematin E (**1**) yellow amorphous powder; [*α*] 25 D − 0.09 (*c* 0.24, MeOH); UV (MeOH) *λ*_max_ (log *ε*) 265 (4.11), 273 (4.14), 312 (3.63), 358 (3.01) nm; IR (KBr) *ν*_max_ 2971, 2914, 1647, 1629, 1584, 1516, 1443, 1397, 1298, 1245, 1127, 979, 925 cm^−1^; ^1^H (500 MHz) and ^13^C (125 MHz) NMR data in CDCl_3_, see Table 1; HR-ESI–MS *m*/*z* 345.1621 [M + H]^+^ (Calcd. for C_20_H_25_O_5_, 345.1621). **1a**: [*α*] 25 D + 24.39 (*c* 0.21, MeOH); ECD (MeOH) *λ*_max_ (Δ*ε*) 214 (+ 5.41), 241 (+ 0.09), 269 (+ 7.51), 297 (− 2.28), 316 (+ 1.71) nm. **1b**: [*α*] 25 D − 25.16 (*c* 0.31, MeOH); ECD (MeOH) *λ*_max_ (Δ*ε*) 203 (− 2.94), 239 (− 0.76), 260 (− 2.75), 295 (+ 2.44), 318 (− 1.20) nm.

**Table 1:** ^1^H (500 MHz) and ^13^C NMR (125 MHz) data of **1** and** 7** (*δ* in ppm, CDCl_3_)

Position	**1**		**7**	
	*δ*_H_ (mlti., *J* in Hz)	*δ*_C_	*δ*_H_ (mlti., *J* in Hz)	*δ*_C_
2	5.35, t (3.5)	100.6	7.84, s	152.3
3	2.99, dd (17.1, 3.7) 2.75, dd (17.1, 3.2)	41.8		123.8
4		194.3		181.2
5		156.3		165.3
6		103.3	6.29, s	95.1
7		159.7		163.4
8		108.9		113.0
9		155.2		153.4
10		102.8		106.3
1ʹ				122.9
2ʹ			7.33, d (7.9)	130.5
3ʹ			6.84, d (8.0)	115.9
4ʹ	6.61, d (10.0)	115.6		156.3
5ʹ	5.48, d (10.0)	125.9	6.84, d (8.0)	115.7
6ʹ		78.1	7.33, d (7.9)	130.5
1ʺ	3.27, dd (14.0, 7.6) 3.20, dd (14.0, 6.8)	21.4		43.9
2ʺ	5.14, m	122.7	4.52, q (6.6)	91.0
3ʺ		131.0	1.50, s	25.8
4ʺ	1.66, s	25.7	1.25, s	21.6
5ʺ	1.78, s	17.8	1.40, d (6.6)	14.3
2-OCH_3_	3.47, s	56.3		
6ʹ-CH_3_	1.44, s 1.42, s	28.4 28.3		

Flemiphilippinin F (**7**) yellow amorphous powder; [*α*]25 D − 1.09 (*c* 0.28, MeOH); ^1^H (500 MHz) and ^13^C (125 MHz) NMR data in CDCl_3_, see Table 1. **7a**: [*α*]25 D + 82.18 (*c* 0.27, MeOH); ECD (MeOH) *λ*_max_ (Δ*ε*) 218 (6.06), 249 (− 2.42), 298 (+ 7.86), 320 (− 1.09) nm. **7b**: [*α*]25 D − 87.15 (*c* 0.31, MeOH); ECD (MeOH) *λ*_max_ (Δ*ε*) 219 (− 0.17), 258 (+ 2.94), 298 (− 7.66), 322, (+ 1.29) nm.

### *A549* and *H1975* cell viability assay

In order to discuss the SAR of the prenyl-substituted chromones and flavonoids and to determine whether the prenyl group is crucial for cytotoxicity against A549 and H1975 cells, the prenyl-substituted chromones and flavonoids (**1** − **7**) isolated from *F. macrophylla* and the commercial non-prenyl analogues (**10** − **13**) were tested. Compounds were dissolved in DMSO and diluted to 5, 10, 20, and 40 μM with RPMI 1640 containing 10% FBS. Fresh culture medium was used as a blank, and icaritin as a positive control. To measure the viability of A549 and H1975 cells, after treatment with the trial compounds, MTT solution (5 mg/mL) was added to test the cytotoxicity of the compounds in vitro. Cells were seeded in a 96-well plate at a density of 5 × 10^3^ cells/well for growth overnight, and then incubated with the trial compounds at different concentrations for 24 h. The original medium in each well was replaced with the MTT reagent diluted 1/10 with RPMI 1640 containing 10% FBS, and the cells were cultured for 4 h under light-protected conditions. The culture medium and cell supernatant were discarded and the cell precipitate was carefully washed to remove impurities from the culture medium. DMSO (100 μL) was added to each well to dissolve the crystalline material completely by shaking on a shaker. Absorbance was measured at 490 nm with a multimode plate reader [20].

### Molecular docking

The RCSB structural protein database (http://www.rcsb.org/pdb) template of p53 (PDB ID: 7B4C) was downloaded. The PyMOL tool was used to remove other homologous peptide chains, water and unrelated ligands in the protein, hydrogen atoms and Gesterg charges were added to prepare protein receptors and its energy was minimized using Chemdraw 3D. Flavonoid binding modes were simulated by AutoDock using blind docking and a grid box 116 × 120 × 126 to cover the entire protein, centred in the middle of the protein (x = 5.664, y = −3.451, z = 9.674). The number of modes was set to 50 and other parameters to default values. The inserted conformational active site was accepted and the lowest binding energy was the preferred conformation. PyMOL software (http://www.pymol.org/) and Discovery Studio were used for visual analysis of parameters (such as binding energy and hydrogen bond distance) based on the docking results [21].

### Cellular thermal shift assay (CETSA)

To confirm that compound **7a** targets p53, a CETSA-WB (Cellular Thermal Shift Assay followed by western blotting) experiment was conducted. After treating with compound **7a** for 24 h, the A549 cells were washed twice with cold PBS. Subsequently, the cells were lysed using RIPA 1640 buffer containing protease or phosphatase inhibitor cocktail. The lysates were centrifuged at 15,000 × g for 20 min at 4 °C. The extracted proteins were divided into 6 equal parts and heated at temperatures of 44, 48, 52, 56, 60, and 64 °C, respectively, for 3 min, followed by a 3 min cooling step to 4 °C. The supernatant was mixed with loading buffer and incubated in microtubes at 100 °C for 7 min. The proteins were then quantified using the western blotting technique [22].

### Western blotting

The cells were lysed by RIPA lysis buffer with 1% cocktail and 1% PMSF and separated by centrifugation. Proteins were extracted from the cell lysates and supernatants. Protein concentration was measured with the BCA protein assay kit. The quantified proteins were separated by SDS-PAGE and then transferred onto the PVDF membrane. The membrane was blocked with 5% non-fat dry milk for 1 h, and was incubated with primary antibody (1:1000 dilution) overnight. The next day, the blot was visualized with HRP-conjugated secondary antibodies (1:5000 dilution). All proteins were visualized with a western blotting substrate. The gray-scale values of related proteins were quantified by ImageJ [23].

## Results and discussion

### Structural elucidation of new compounds 1 and 7

**Fig. 2:**
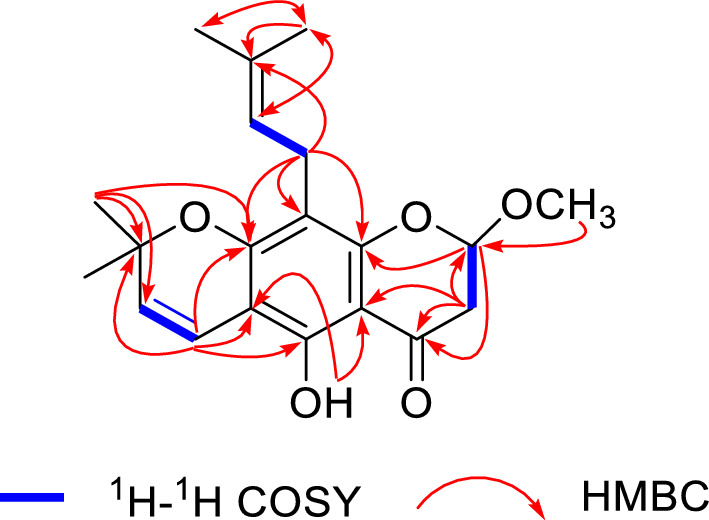
^1^H-.^1^H COSY and selected HMBC correlations of compound** 1**

**Fig. 3:**
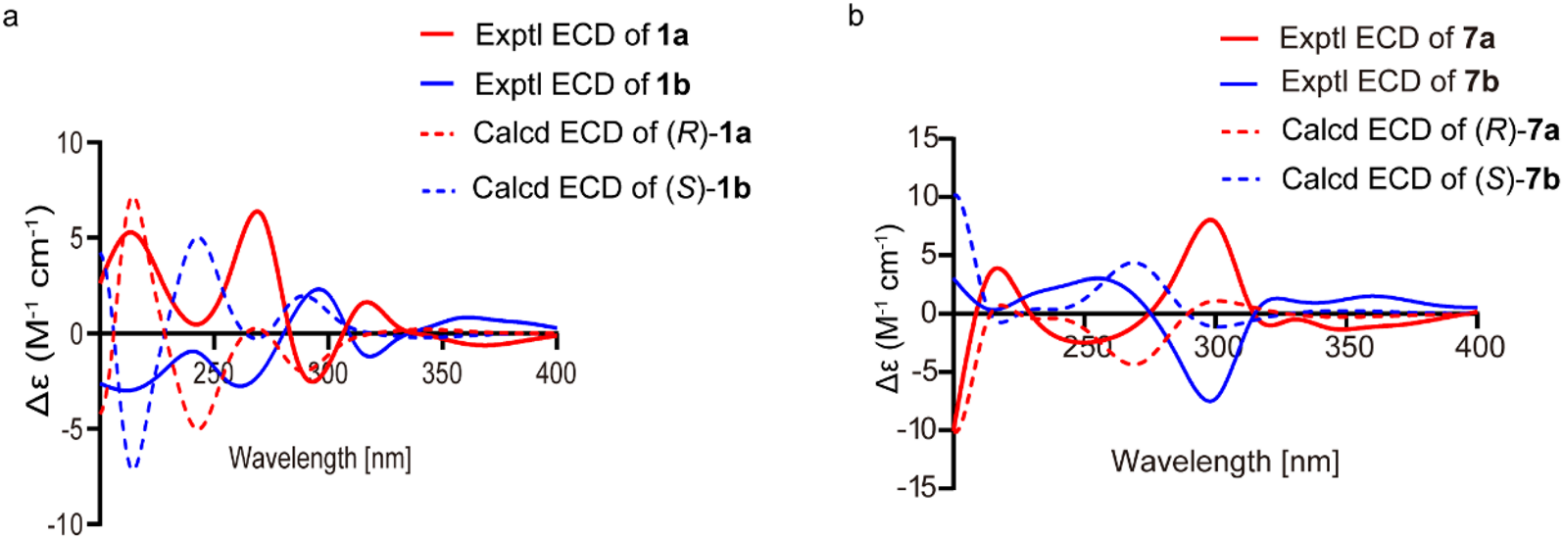
Experimental and calculated ECD spectra of **1a**/**1b** (**a**) and **7a**/**7b** (**b**)

Compound **1** was obtained as a yellow amorphous powder with [*α*]25 D − 0.09 (*c* 0.24, MeOH). Its molecular formula C_20_H_24_O_5_, with nine indices of hydrogen deficiency, was assigned from HR-ESI–MS *m*/*z* 345.1621 [M + H]^+^ (Calcd. for C_20_H_25_O_5_, 345.1621). The IR spectrum showed absorption bands characteristic of olefinic bond (1647 cm^−1^), conjugated carbonyl (1629 cm^−1^), and aromatic (1584, 1516, and 1443 cm^−1^) functionalities. The UV spectrum showed absorption maxima at 265, 273, 312, and 358 nm, indicating that **1** was a pyranochromanone derivative [24]. The ^1^H NMR spectrum (Table 1) displayed signals of a chromanone moiety at *δ*_H_ 5.35 (1H, t, *J* = 3.5 Hz), 2.99 (1H, dd, *J* = 17.1, 3.7 Hz), 2.75 (1H, dd, *J* = 17.1, 3.2 Hz), a chelated hydroxy group at *δ*_H_ 12.15 (1H, s), a *gem*-dimethylpyran moiety at *δ*_H_ 6.61 (1H, d, *J* = 10.0 Hz), 5.48 (1H, d, *J* = 10.0 Hz), 1.44 (3H, s), and 1.42 (3H, s), a prenyl group at *δ*_H_ 5.14 (1H, m), 3.27 (1H, dd, *J* = 14.0, 7.6 Hz), 3.20 (1H, dd, *J* = 14.0, 6.8 Hz), 1.78 (3H, s), and 1.66 (3H, s), and an oxygenated methyl at *δ*_H_ 3.47 (s). The ^13^C NMR spectrum (Table 1) in combination with 2D NMR spectra (Fig. 2) resolved 20 carbon signals ascribed to one keto carbonyl at *δ*_C_ 194.3, one acetal methine at *δ*_C_ 100.6, one methylene adjacent to carbonyl group at *δ*_C_ 41.8, one benzene ring at *δ*_C_ 156.3, 103.3, 159.7, 108.9, 155.2, and 102.8, one gem-dimethylpyran moiety at *δ*_C_ 115.6, 125.9, 78.1, 28.4, and 28.3, one prenyl group at *δ*_C_ 21.4, 112.7, 131.0, 25.7, and 17.8, and one oxygenated methyl at *δ*_C_ 56.3. The HMBC correlations from O*H* to C-5/C-6/C-10 indicated that the chelated hydroxy group was positioned at C-5. The HMBC correlations from H-4ʹ to C-5/C-6/C-7/C-6ʹ, and from 6ʹ-C*H*_3_ to C-7/C-5ʹ established the attachment of the *gem*-dimethylpyran moiety to C-6/C-7 of the chromanone moiety. The HMBC correlations from H_2_-1ʺ to C-7/C-8/C-9/C-3ʺ suggested that the prenyl group was attached to C-8. The above evidence indicated that **1** was a prenylated *gem*-dimethylpyranochromanone with an additional methyl group compared to eriosematin E [25], which was supported by the HMBC correlations from H-2 to C-4/C-9, and from 2-OC*H*_3_ to C-2 (Fig. 2). Consequently, the structure of **1** was identified as 5-hydroxy-2-methoxy-6ʹ,6ʹ-dimethylpyrano(2ʹ,3ʹ:7,6)-8-(3ʺ,3ʺ-dimethylallyl) chromanone, and named 2-*O*-methyl eriosematin E. However, the ECD spectrum of **1** showed no Cotton effect, suggesting that **1** was a racemic mixture. Subsequently, **1** was separated by chiral HPLC using a Hplcone CHiRALONE 5-C column, yielding a pair of enantiomers 1a and 1b whose ECD spectra revealed opposite Cotton effects (Fig. 3a) and optical rotations (**1a**: [*α*] 25 D + 24.39; **1b**: [*α*] 25 D − 25.61). The absolute configurations of the two enantiomers were determined by comparison of the experimental ECD spectra with those calculated using time-dependent density functional theory [26]. The experimental ECD spectra of **1a** and **1b** corresponded well with those calculated for (*R*)-**1a** and (*S*)-**1b** (Fig. 3a), respectively. Thus, the structures of **1a** and **1b** were designated as (*R*)- and (*S*)-2-O-methyl eriosematin E, respectively.

Compound **7** was isolated as a yellow powder. Its ^1^H and ^13^C NMR data (Table 1) were identical to those of flemiphilippinin F [27], indicating that **7** had the same planar structure as flemiphilippinin F. However, in previous reports [27–29], the configuration of the chiral center at C-2ʺ was not determined. Due to the near-zero optical rotation value of [*α*]25 D − 1.09 (*c* 0.28, MeOH), compound **7** was assumed to be a racemate. Chiral HPLC resolution of **7** afforded a pair of enantiomers **7a** ([*α*]25 D + 82.18) and **7b** ([*α*]25 D − 87.15). By comparing the experimental and calculated ECD spectra (Fig. 3b), the absolute configurations of **7a** and **7b** were assigned as (2ʺ*R*) and (2ʺ*S*), respectively. Therefore, compounds **7a** and **7b** were established as ( +)-(2ʺ*R*)-flemiphilippinin F and ( −)-(2ʺ*S*)-flemiphilippinin F, respectively.

The other known compounds, eriosemaone A **2** [25], khonklonginol G **3** [30], 6,8-diprenyl-4-methyl-naringenin **4** [31], khonklonginol H **5** [30], derrone **6** [32], juniper camphor **8** [33], and (24*S*)-stigmasterol-4-en-3-one **9** [34] were identified by comparing the NMR and MS data with those in the literature.

A549 cells are widely used in vitro in type II lung epithelial cell models for drug metabolism studies [35]. Cytotoxicity against A549 cells of compounds **1** − **7**, and four analogues **10** − **13** (Fig. 1) were evaluated. As shown in Table 2, compounds **5** − **7** displayed potent cytotoxicity against A549 cells with IC_50_ values of 4.50 − 19.83 μmol·L^−1^, compared with 74.24 μmol·L^−1^ for the positive control icaritin. All compounds showed enhanced cytotoxicity against H1975 compared with A549 cells, among which compounds 1b, 5 – 7 was the most toxic with an IC50 value of less than 5 μmol·L^-1^. Moreover, in the same cell type, there was no significant difference between the enantiomers 7a and 7b in cytotoxicity, while the cytotoxicity of enantiomer 1a was lower than 1b.

**Table 2:** Cytotoxicity against A549 and H1975 cells of compounds 1 − 7, and 10 − 13

Compd.	24h IC_50_ (μmol·L^-1^)	
	A549 cells	H1975 cells
1a	79.89 ± 3.66	21.42 ± 1.22***
1b	49.27 ± 1.81***	< 5***
2	36.59 ± 1.01***	20.25 ± 0.65***
3	43.49 ± 3.62***	28.46 ± 0.53***
4	> 100	42.77 ± 1.60***
5	4.50 ± 0.87***	< 5***
6	19.83 ± 0.78***	< 5***
7a	19.25 ± 0.13***	< 5***
7b	19.07 ± 0.07***	< 5***
10	> 100	> 100
11	> 100	> 100
12	> 100	46.32 ± 0.85***
13	> 100	> 100
Icaritin^b^	74.24 ± 1.46	15.16 ± 0.18

^*a*^Each data is shown as the mean ± SD calculated from three replicate determinations

^*b*^Positive control

^***^P < 0.001 versus the blank control group

### Structure–activity relationship analysis in A549 cells

**Fig. 4:**
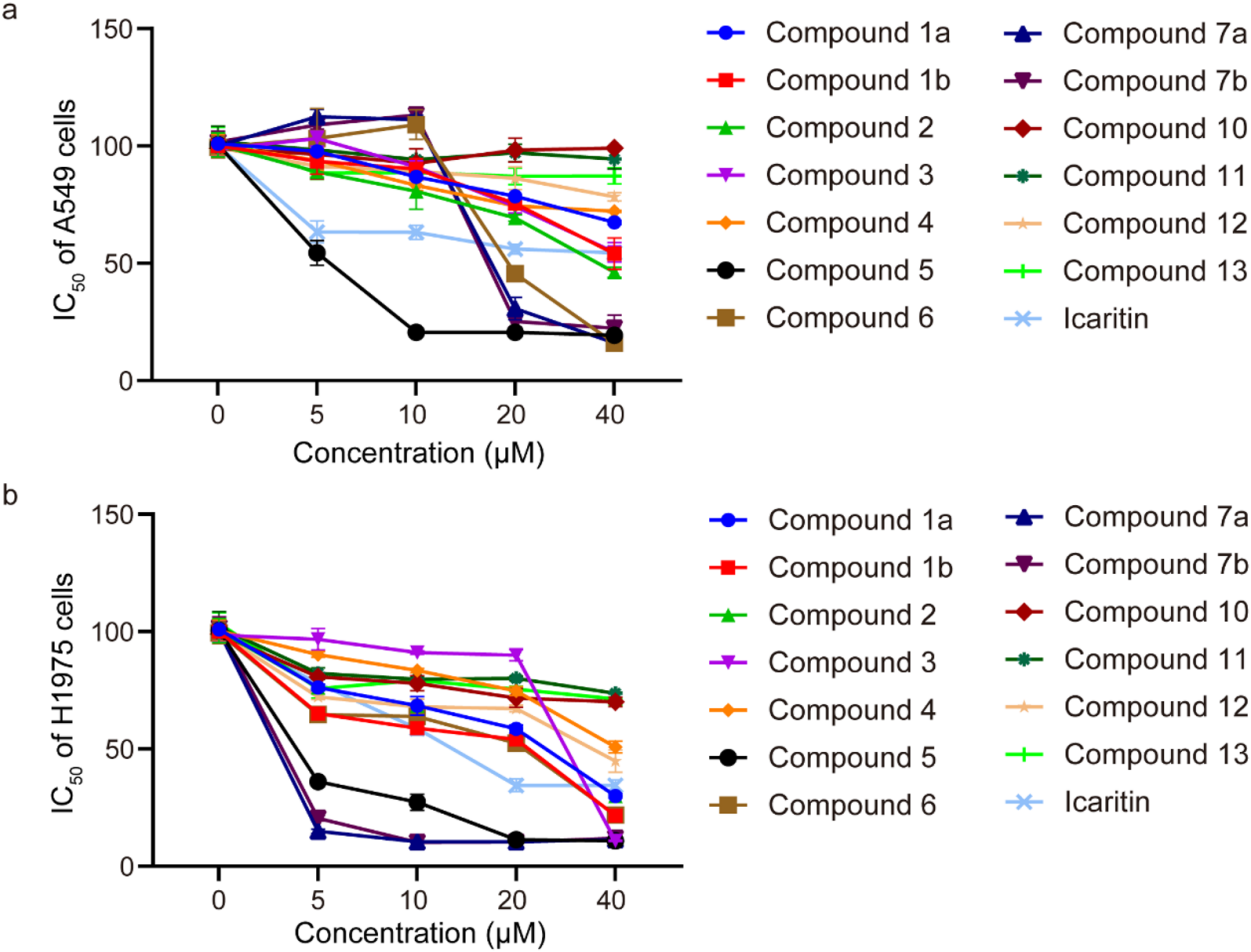
Cell viability with different compounds (icaritin as positive control) at different concentrations, with at least three replicates for each data point. **a** Different concentrations, the survival of A549 cells in different compounds. **b** Different concentrations, the survival of H1975 cells in different compounds

It has been reported that prenyl (3-methyl-2-butenyl or isopentenyl) substitution on a flavonoid’s aromatic ring can enhance affinity to P-glycoprotein and permeability to cell membranes, and significantly improve their bioactivity [36]. Considering that the activities of prenylated chromones and flavonoids (**1** − **7**) were stronger than those of non-prenyl chromones and flavonoids (**10** − **13**) (Table 2 and Fig. 4), it could be concluded that the prenyl substituent was favorable for cytotoxicity against A549 cells. Among the prenylated chromones and flavonoids (**1** − **7**), compound **4** was the weakest (Table 2), indicating that the cyclization of the 6/8-prenyl group with 7-OH is crucial for the activity. Specifically, compared to compound **4** which bears two prenyl groups at C-8 and C-6, the γ,γ-dimethylpyran ring formed by the prenyl at C-6/8 and the hydroxy group at C-7 (**3**,** 5**, and **6**) significantly enhanced inhibition of A549 cells. This indicates that the number of prenyl groups had little effect on the activity, but that their structural form was significant, especially in the γ,γ-dimethylpyran ring form. Moreover, compared to compound **6** that had a γ,γ-dimethylpyran ring formed by the prenyl at C-8 and the hydroxy at C-7, the 2-methyl-3,3-dimethyl tetrahydrofuran ring formed by the prenyl at C-8 and the hydroxy at C-7 (**7a** and **7b**) increased cytotoxicity, indicating higher activity of the 5-membered than the 6-membered ring. The SAR described above indicated that the prenyl group was essential for chromones and flavonoids to exert toxicity on A549 cells, and that prenyl in cyclized form could enhance activity, especially in the 2-methyl-3,3-dimethyl tetrahydrofuran form. It is worth noting that compounds **1a**, **1b**, and **2**, which lack the B-ring, had lower activity against A549 cells than compounds **5** − **7** which bear a B-ring substituted at C-2 or C-3. Comparing the activities of non-prenyl chromones and flavonoids **10** − **13** (Fig. 4), it was found that the activity sequence was **12** > **13** > **11** > **10** (flavonoids > chromones), indicating that the B-ring of the flavonoids was positively associated with cytotoxic activity against A549 cells. Notably, compared to compound **3**, introduction of a 2ʹ-OH substituent (**5**) increased the cytotoxic activity against A549 cells. Compared to (*R*)-**1a**, (*S)-***1b** exhibited higher cytotoxic activity, indicating that the chirality at C-2 significantly affected cytotoxic activity against A549 cells. Meanwhile, comparing 7a to 7b, chirality at C-2ʺ appears to have little impact on the toxicity against A549 cells.

**Fig. 5:**
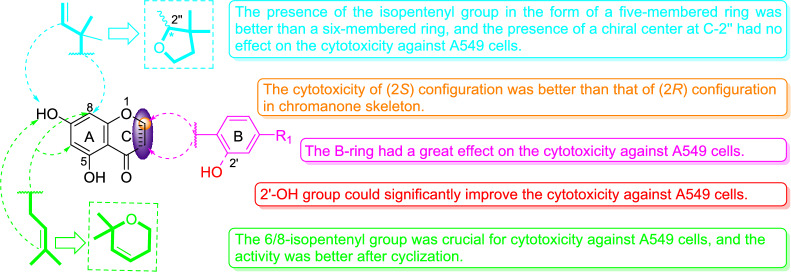
Summary of the SAR of chromones and flavonoids

In conclusion, the increase in activity due to prenyl substitution was not related to the number of prenyl groups, but rather to the position, form, B-ring substitution, and parent structure. The cyclization of the 6/8-prenyl group with 7-OH was crucial for the activity, and the B-ring and 2ʹ-OH substitution increased cytotoxicity. Based on these results of the inhibitory effects of chromones and flavonoids on A549, the SAR of these compounds is summarized in Fig. 5.

### Molecular docking on p53 protein

The inhibition mechanism was confirmed from the docking data summarized in Table 3 and analysis with AutoDock, PyMOL and Discovery Studio. The absolute binding energies between p53 and the flavonoids are ranked as compounds **5** > **7a** = **7b** > **6** = **3** > **13** > **2** > **11** > icaritin > **4** > **1b** > **1a** > **12** > **10**. To further investigate the interaction between P53 and the flavonoids, the molecular docking results are summarized in Additional file 1: Figs. S18 − S20. The results show that there are about nineteen amino acid residues around the chromones (Ser121, Val122, Thr123, Cys124, Ser116, His115, Leu114, Ala119, Ala161, Ala159, Ili195, Tyr205, His193, Leu194, Gln192, Val173, His214, Arg174, Val216), twelve amino acid residues around the chromanones (Cys124, Val122, Ser121, Thr125, Thr123, Tyr126, Phe113, Leu114, His115, Ser116, Pro128, Arg128) (Additional file 1: Fig. S18), sixteen amino acid residues around the flavanones (Thr123, Cys124, Thr140, Pro142, Leu114, Val122, Ser116, Ala119, Thr231, Ser227, Cys229, Pro222, Glu224, Glu221, Pro219, Asn200) (Additional file 1: Fig. S19), twelve amino acid residues around the isoflavones (Ala119, Thr118, Arg283, Pro278, Val122, Gly279, Arg280, Lys120, Cys277, Gln136, Thr123, Ala276), and seven amino acid residues around icaritin (Cys124, Thr123, Val122, Thr140, Pro142, Leu114, Ser116) (Additional file 1: Fig. S20). These amino acid residues were bound to the flavonoids through hydrogen bonding, van der Waals, π-sulfur, π-π stacking, or π-alkyl interactions. The selected prenyl chromones or flavonoids can enter the active site of p53 as activators and form hydrogen bonds, alkyl, and π-alkyl interactions with amino acid residues. In addition, different types of chromones or flavonoids bound to different amino acid residues, indicating that different chromones or flavonoids could be non-competitive activators of p53. Except for compound **1**, compared to their parent structure, the prenyl chromones or flavonoids showed significantly increased binding energy and activity with p53 without an increase in the number of hydrogen bonds. This would indicate that alkyl and π-alkyl interactions were key driving forces for the binding of prenyl flavonoids to p53.

**Table 3:** Molecular docking results of p53 with flavonoids

Compd	Interactions (Amino acid residues lining the binding site, distance and binding energy)			
	Key residues	π-π stacked	Hydrogen binding	Eb
**1a**	CYS124, LEU114, HIS115, VAL122, SER121, SER116, THR123, ALA119	VAL122 (4.50)	SER121 (2.62) THR123 (3.01)	−7.74
**1b**	CYS124, LEU114, HIS115, VAL122, SER121, SER116, THR123, ALA119	VAL122 (4.22)	SER121 (2.15)	−7.77
**2**	LEU114, SER116, PRO142, CYS124, VAL122, THR123, SER121	VAL122 (4.83, 3.28)	SER116 (2.14) CYS124 (4.03)	−8.42
**3**	THR123, CYS124, THR140, PRO142, LEU114, VAL122, SER116, ALA119	VAL122 (4.95) ALA119 (3.81) SER116 (4.76)	SER116 (2.14) CYS124 (4.03)	−8.44
**4**	LEU114, SER116, THR140, CYS124, VAL122, PRO142, THR123, ALA119	VAL122 (4.86) ALA119 (4.94) SER116 (4.79) CYS124 (5.77)	SER116 (1.75) CYS124 (3.88) THR123 (2.87, 2.74)	−8.17
**5**	LEU114, SER116, ALA119, VAL122, THR123, CYS124, PRO142, THR140	ALA119 (5.37) VAL122 (4.98) CYS124 (5.32) PRO142 (5.45)	CYS124 (3.41) THR123 (2.50)	−9.0
**6**	ALA119, THR118, ARG283, PRO278, VAL122, GLY279, ARG280, LYS120, CYS277	PRO278 (4.91) ARG280 (3.69, 5.40) CYS277 (3.70, 3.84) LYS120 (2.83, 3.96, 4.63)	VAL122 (3.33) ARG280 (1.96) GLY279 (1.77, 2.39)	−8.68
**7a**	CYS277, LYS120, GLN136, PRO278, THR123, VAL122, GLY279, ARG280, ARG283, THR118, ALA119	PRO278 (4.80) ARG280 (3.67, 5.40) CYS277 (3.69, 4.00) LYS120 (4.62, 3.90,2.82)	ARG280 (1.95) GLY279 (2.34, 1.70) VAL122 (3.24) GLN136 (2.95)	−8.83
**7b**	CYS277, LYS120, GLN136, PRO278, THR123, VAL122, GLY279, ARG280, ARG283, THR118, ALA119	PRO278 (4.80) ARG280 (3.67, 5.40) CYS277 (3.69, 4.00) LYS120 (4.62, 3.90, 2.82)	ARG280 (1.95) GLY279 (2.34, 1.70) VAL122 (3.24) GLN136 (2.95)	−8.83
**10**	ARG282, HIS115, PRO128, THR125, PHE113, LEU114, TYR126, SER116	VAL216 (5.48) ILE195 (4.05)	ALA161 (1.64) HIS214 (2.12) LEU194 (1.71)	−6.63
**11**	ALA161, ILE195, ALA159, TYR205, HIS193, LEU194, HIS214, GLN192, ARG174, VAL173, VAL216	ARG282 (3.96) PRO128 (5.22)	THR125 (2.99) LEU114 (1.75) TYR126 (1.94)	−8.33
**12**	ASN200, PRO219, GLU221, GLU224, PRO222, CYS229, SER227, THR231		PRO219 (1.95) ASN200 (1.75) GLU224 (2.47) CYS229 (2.13)	−7.23
**13**	ALA276, LYS120, CYS277, PRO278, VAL122, GLY279, ARG280, ARG283, THR118, ALA119	PRO278 (4.83) CYS277 (3.41, 3.80) ARG280 (3.78, 5.21) LYS120 (4.66, 3.91, 2.94)	ARG280 (1.92) VAL122 (3.37) GLY279 (2.33, 1.73)	−8.22
**icaritin**	CYS124, THR123, VAL122, THR140, PRO142, SER116, LEU114	LEU114 (4.44) SER116 (4.07) VAL122 (3.22,5.43)	CYS124 (3.65) THR123 (2.59)	−8.53

The prenyl groups in the form of γ,γ-dimethylallyl mostly participate in alkyl and π-alkyl interactions with Leu114 of the p53 protein. Flavonoids or chromanones with different numbers of hydroxy groups form hydrogen bonds with different amino acid residues of p53, and their activity is also different. Therefore, it is speculated that hydrogen bonding is another major interaction mode for activation of p53 by flavonoids or chromones. A comparison of the docking results showed that the binding position of Cys124 changed with slight structural changes. In compounds 1 − 3, 5, and 6, the prenyl groups at C-6 in the form of γ,γ-dimethylpyran rings bind to the Cys124 residue. It is noteworthy that changing the C-2 OMe configuration changes the binding positions of compounds **1a** (2-OMe binding with Cys124) and **1b** (γ,γ-dimethylpyran ring binding with Cys124) (Additional file 1: Fig. S18), resulting in significant differences in activity. Furthermore, **1b** has more binding with Cys124 than **1a**, so the activity of **1b** is significantly increased. The interactions of compounds **1** and **2** with Cys124 are mainly alkyl and π-alkyl. In addition, compound **11** was inactive despite its high binding energy, perhaps due to interactions outside the active site of p53 protein.

As shown in Additional file 1: Fig. S19, the mechanism of flavanone binding to p53 was elucidated. Prenyl-substituted flavanones bound amino acid residues different from those of compound **12**. In compound **4**, the prenyl groups at C-8 and C-6 exist as γ,γ-dimethylallyl, and the C-5 hydroxy forms a hydrogen bond with Cys124. In compound **3**, the prenyl group at C-6 exists as a γ,γ-dimethylpyran ring, and the oxygen atom in the pyran ring forms a hydrogen bond with Cys124. In compound **5**, the hydroxy group at C-2ʹ of the B-ring leads to fewer hydrogen bonds in its p53 complex compared to the complexes of compounds **3** and **4**, but the activity is significantly increased. A possible explanation for this is that when the prenyl group exists as γ,γ-dimethylallyl at the C-6/8 of the A-ring, the steric hindrance of the C-7 hydroxy is increased. In contrast, when the C-7 hydroxy cyclizes with the prenyl group, steric hindrance is reduced. When the hydroxy group is at the B-ring it also has a smaller steric hindrance and the O atom electron cloud density increases, facilitating hydrogen bond formation with Cys124. Cys124 is a key amino acid residue for the activity of flavanones and prenyl binding to this residue may be an important mechanism for their activity. However, the amino acid residue for such an interaction has not been reported, and whether it is a new active site needs further verification.

It has been reported that Cys277 is an amino acid residue that small molecule PK11000 specifically alkylates to bind with p53 [37], increasing the stability of the p53 protein. Compounds 6 and 7 form π-sulfur interactions with Cys277 (Additional file 1: Fig. S20), so they may have a therapeutic effect similar to PK11000. Compound **7** has a 2-methyl-3,3-dimethyltetrahydrofuran ring, while compound **6** has a γ,γ-dimethylpyran ring. The double bond in the tetrahydrofuran ring is reduced, increasing the electron density of the O atom’s outer electron cloud, facilitating hydrogen bond formation and increasing the binding energy and activity. In addition, compounds **7a** and **7b** bind to p53 in the same pattern, which is consistent with the activity test results.

The hydroxy group at position C-3 of icaritin does not form a hydrogen bond with any amino acid residue, reducing the binding energy. The binding of the C-7 hydroxy with Cys124 confirms that this is a crucial amino acid residue for activity.

In summary, flavonoids or chromones with a prenyl substituent can bind to the p53 protein, but different amino acid residues may result in different activity levels. The prenyl in cyclic form can enhance the stability of the flavonoid or chromone-p53 complex. Hydroxy groups on the B-ring affect the binding energy and activity. Different parent prenyl flavonoids may have different binding energies due to differences in their spatial configuration. Overall, these results illustrate the p53 binding mechanism for prenyl chromones or flavonoids, suggesting that their activity is exerted through the key residues Cys124 and Cys277. The results provide a reference for further studies on the optimal conformation of biologically active prenyl chromones or flavonoids and their interaction with p53.

### Compound 7a induces apoptosis in A549 cells by regulating the activity of p53 protein

**Fig. 6:**
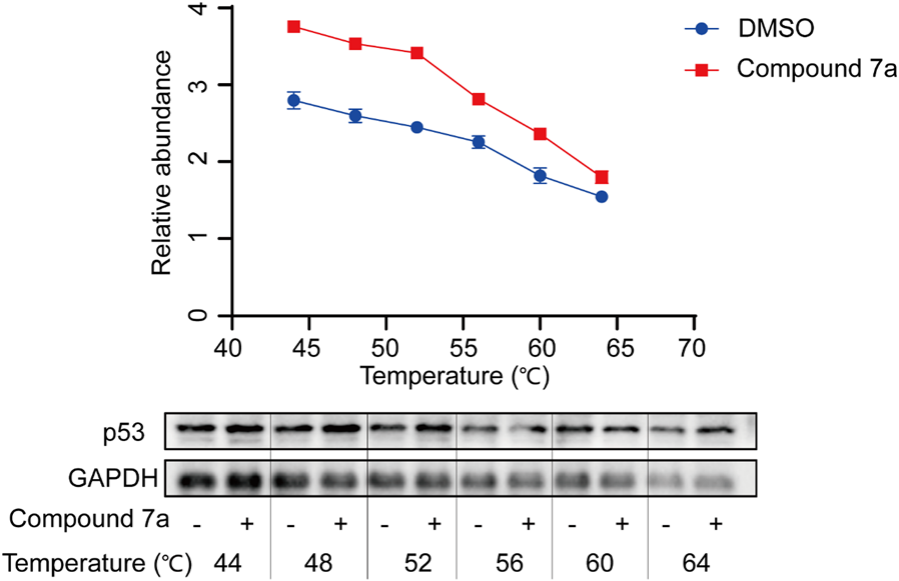
CETSA-WB experiment to confirm that compound **7a** targeted p53 proteins

**Fig. 7:**
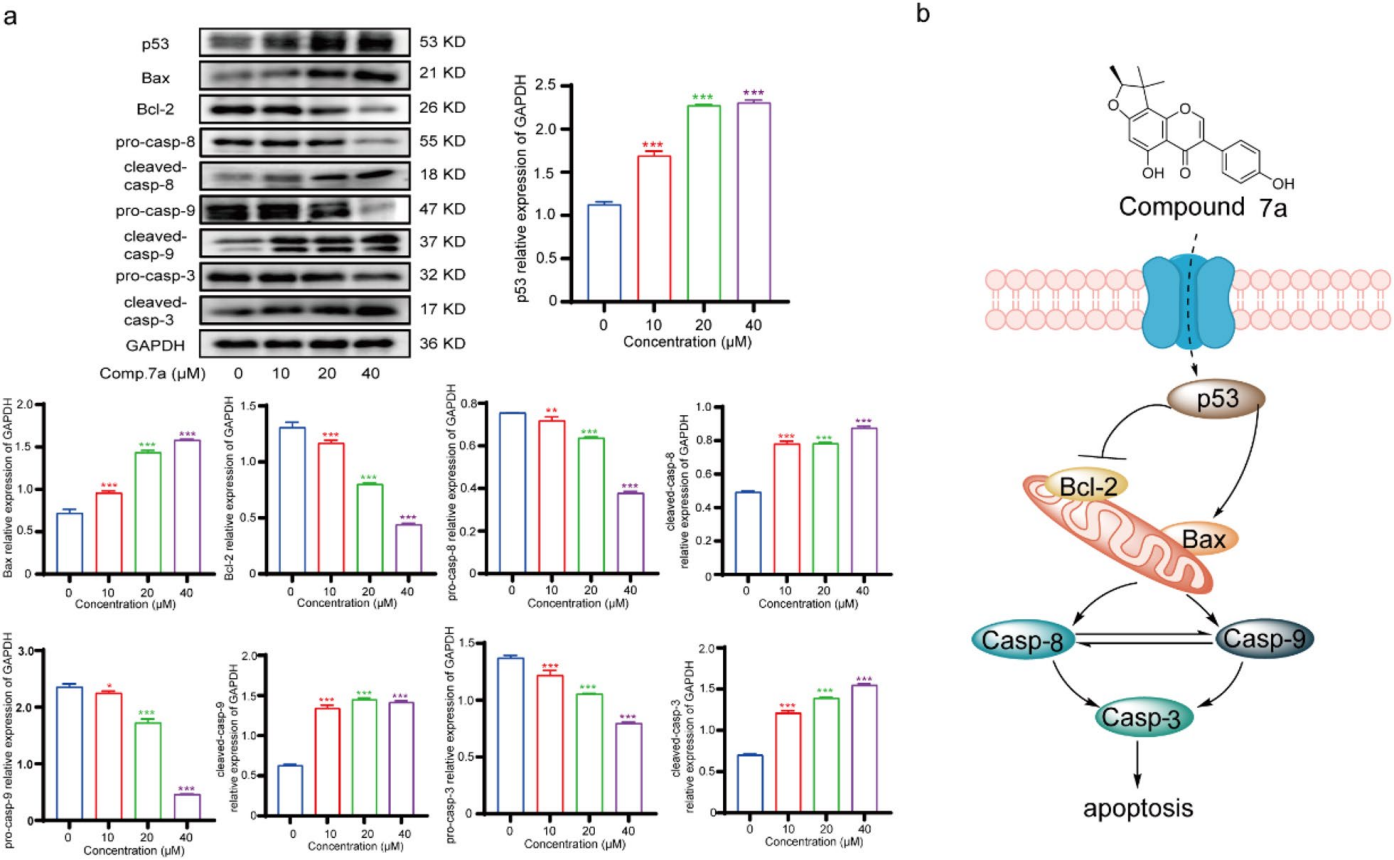
**a** The effect of compound 7a on the expression of apoptosis-related enzymes in A549 cells. A549 cells were treated with 7a at 40 μmol/L for 24 h. Data are represented as mean ± SD (n = 3). **P < 0.01, ***P < 0.001 versus the blank control group. **b** Compound 7a induced A549 cell apoptosis by activating the p53 protein

We further confirmed the interaction between compound **7a** and p53 through CETSA-WB experiments. A549 cells were treated with compound **7a** (40 μM) or DMSO and underwent CETSA thermal pulse treatment. As shown in Fig. 6, the thermal stability of p53 was significantly increased in the **7a**-treated group. To further clarify the mechanism of prenyl flavonoid-induced A549 cell toxicity, we detected p53 protein and apoptosis-related proteins, as shown in Fig. 7a. In A549 cells treated with compound **7a**, the expression of p53 protein increased, activating Bax expression and increasing the expression of Bax protein, thus competitively reducing the expression of Bcl-2. Bcl-2 protects cells from apoptosis by inhibiting the activation of caspase enzymes. Therefore, reduced Bcl-2 expression led to a decrease in the inhibitory effect on caspase enzyme activity. Herein, we observed a significant decrease in the expression of Pro-Casp8, Pro-Casp9, and Pro-Casp3 in cells relative to the blank group, with a corresponding significant increase in the expression of cleaved-Casp8, cleaved-Casp9, and cleaved-Casp3 relative to the blank group. These data indicate that compound **7a** directly acts on the p53 protein, activating the Bax protein, increasing the expression of cleaved-Casp8, cleaved-Casp9, and cleaved-Casp3, and promoting the cytotoxic apoptosis of A549 cells (Fig. 7b).

## Conclusions

In this study, a series of prenylated chromones and flavonoids isolated from *F. macrophylla* were shown to exhibit anticancer activity in A549 cells. SAR studies demonstrated that the cyclization of the 6/8-prenyl group with 7-OH was essential for cytotoxicity against A549 cells, and the B-ring and 2ʹ-OH substitution significantly enhanced the activity. Molecular docking revealed the binding mechanism of the prenyl chromones or flavonoids with the p53 protein, which was further validated by CETSA. It was demonstrated that prenyl chromones or flavonoids might induce apoptosis in A549 cells through the p53 pathway.

### Supplementary Information

**Additional file 1: Section S1.** ECD calculation. **Table S1.** Standard orientation of (*R*)-**1a-**aat B3LYP/6-31G(d) level in gas phase. **Table S2.** Standard orientation of (*R*)-**1a-**bat B3LYP/6-31G(d) level in gas phase. **Table S3.** Standard orientation of (*R*)-**7a-**aat B3LYP/6-31G(d) level in gas phase. **Table S4.** Standard orientation of (*R*)-**7a-**bat B3LYP/6-31G(d) level in gas phase. **Table S5.** Standard orientation of (*R*)-**7a-**cat B3LYP/6-31G(d) level in gas phase. **Figure S1.** ( +) HR-ESI–MS sepectrum of compound **1. Figure S2.** IR spectrum of compound **1. Figure S3.** UV spectrum of compound **1** in MeOH. **Figure S4.** ^1^H NMR sepectrum of compound **1** in CDCl_3_. **Figure S5.** ^13^C NMR sepectrum of compound **1** in CDCl_3_. **Figure S6.** DEPT-135 spectrum of compound **1** in CDCl_3_. **Figure S7.** ^1^H-^1^H COSY spectrum of compound **1** in CDCl_3_. **Figure S8.** HSQC spectrum of compound **1** in CDCl_3_. **Figure S9.** HMBC spectrum of compound **1** in CDCl_3_. **Figure S10.** Chiral HPLC analysis of compound **1. Figure S11.** ECD spectrum of compound **1a** in MeOH. **Figure S12.** ECD spectrum of compound **1b** in MeOH. **Figure S13.** 1H NMR sepectrum of compound **7** in CDCl3. **Figure S14.** 13C NMR sepectrum of compound **7** in CDCl_3_. **Figure S15.** Chiral HPLC analysis of compound** 7**. **Figure S17** ECD spectrum of compound **7b** in MeOH. **Figure S18.** The molecule docking results of chromones and chromanones with p53. **Figure S19.** The molecule docking results of flavanones with p53. **Figure S20.** The molecule docking results of isoflavones and icaritin with p53.

## Data Availability

All data generated or analyzed during this review is included in published articles.
