# Supplementary material for: Prenylated chromones and flavonoids isolated from the roots of Flemingia macrophylla and their anti-lung cancer activity
Source: Chin Med. 2023 Nov 23;18:153. doi: 10.1186/s13020-023-00860-3 (PMC10668522; doi:10.1186/s13020-023-00860-3)
Supplement: Supplementary file 1 — Additional file 1: Section S1. ECD calculation. Table S1. Standard orientation of (R)-1a-aat B3LYP/6-31G(d) level in gas phase. Table S2. Standard orientation of (R)-1a-bat B3LYP/6-31G(d) level in gas phase. Table S3. Standard orientation of (R)-7a-aat B3LYP/6-31G(d) level in gas phase. Table S4. Standard orientation of (R)-7a-bat B3LYP/6-31G(d) level in gas phase. Table S5. Standard orientation of (R)-7a-cat B3LYP/6-31G(d) level in gas phase. Figure S1. ( +) HR-ESI–MS sepectrum of compound 1. Figure S2. IR spectrum of compound 1. Figure S3. UV spectrum of compound 1 in MeOH. Figure S4. 1H NMR sepectrum of compound 1 in CDCl3. Figure S5. 13C NMR sepectrum of compound 1 in CDCl3. Figure S6. DEPT-135 spectrum of compound 1 in CDCl3. Figure S7. 1H-1H COSY spectrum of compound 1 in CDCl3. Figure S8. HSQC spectrum of compound 1 in CDCl3. Figure S9. HMBC spectrum of compound 1 in CDCl3. Figure S10. Chiral HPLC analysis of compound 1. Figure S11. ECD spectrum of compound 1a in MeOH. Figure S12. ECD spectrum of compound 1b in MeOH. Figure S13. 1H NMR sepectrum of compound 7 in CDCl3. Figure S14. 13C NMR sepectrum of compound 7 in CDCl3. Figure S15. Chiral HPLC analysis of compound 7. Figure S17 ECD spectrum of compound 7b in MeOH. Figure S18. The molecule docking results of chromones and chromanones with p53. Figure S19. The molecule docking results of flavanones with p53. Figure S20. The molecule docking results of isoflavones and icaritin with p53. [file 13020_2023_860_MOESM1_ESM.pdf]

Supplementary Material

Prenylated chromones and flavonoids isolated from the roots of *Flemingia macrophylla* and their anti-tumor activity against A549 cells

Baolin Wang [1 Δ], Qinqin Wang [2 Δ], Renyikun Yuan[1, 3], Shilin Yang[2, 3], Meilin Lu[1], Fuhong Yuan[1], Zhidan Dong[1], Menghuan Mo[1], Qiming Pan[1*], Hongwei Gao[1, 3*]

[1] College of Pharmacy, Guangxi University of Chinese Medicine, Nanning 530200, China;

[2] College of Pharmacy, Jiangxi University of Traditional Chinese Medicine, Nanchang 330004, China;

[3] Guangxi Engineering Technology Research Center of Advantage Chinese Patent Drug and Ethnic Drug Development, Nanning 530020, China

[Research funding] The work was supported financially by National Key Research and Development Program of China (2019YFC1719304) and Jiangxi Province 2023 Annual college students Innovation and Entrepreneurship Training plan (S202310412094).

[Δ] Their contributions to the article were consistent.

[* Corresponding authors] *E-mail addresses*: qmingpan@163.com (PAN Qi-Ming), gaohongwei06@126.com (GAO Hong-Wei).

List Of Contents

S1. ECD calculation 3
Table S1 Standard orientation of (*R*)-**1a**-a at B3LYP/6-31G(d) level in gas phase 3
Table S2 Standard orientation of (*R*)-**1a**-b at B3LYP/6-31G(d) level in gas phase 4
Table S3 Standard orientation of (*R*)-**7a** -a at B3LYP/6-31G(d) level in gas phase 4
Table S4 Standard orientation of (*R*)-**7a** -b at B3LYP/6-31G(d) level in gas phase 5
Table S5 Standard orientation of (*R*)-**7a**-c at B3LYP/6-31G(d) level in gas phase 6
Fig. S1 (+) HR-ESI-MS sepectrum of compound **1** 9
Fig. S2 IR spectrum of compound **1** 9
Fig. S3 UV spectrum of compound **1** in MeOH 10
Fig. S4 ^{1}H NMR sepectrum of compound **1** in $CDCl_3$ 10
Fig. S5 ^{13}C NMR sepectrum of compound **1** in $CDCl_3$ 11
Fig. S6 DEPT-135 spectrum of compound **1** in $CDCl_3$ 11
Fig. S7 ^{1}H-^{1}H COSY spectrum of compound **1** in $CDCl_3$ 12
Fig. S8 HSQC spectrum of compound **1** in $CDCl_3$ 12
Fig. S9 HMBC spectrum of compound **1** in $CDCl_3$ 13
Fig. S10 Chiral HPLC analysis of compound **1** 13
Fig. S11 ECD spectrum of compound **1a** in MeOH 14
Fig. S12 ECD spectrum of compound **1b** in MeOH 14
Fig. S13 ^{1}H NMR sepectrum of compound **7** in $CDCl_3$ 15
Fig. S14 ^{13}C NMR sepectrum of compound **7** in $CDCl_3$ 15
Fig. S15 Chiral HPLC analysis of compound **7** 16
Fig. S16 ECD spectrum of compound **7a** in MeOH 16
Fig. S17 ECD spectrum of compound **7b** in MeOH 17
Fig. S18 The molecule docking results of chromones and chromanones with p53 18
Fig. S19 The molecule docking results of flavanones with p53 19
Fig. S20 The molecule docking results of isoflavones and icaritin with p53 20

S1. ECD calculation

The CONFLEX [1] searches based on molecular mechanics with MMFF94S force fields were performed for compounds (*R*)-**1a**, (*R*)-**7a**. Selected conformers with distributions higher than 1% were further optimized by the density functional theory method at the B3LYP/6-31G* level in Gaussian 09 program package [2], leading to stable geometries (ΔE < 2 kcal/mol), which were in good agreement with the ROESY data. The optimized geometries were further checked by frequency calculation and resulted in no imaginary frequencies. The ECD was calculated using TD-DFT-B3LYP/6-31G+(d, p) of theory on B3LYP/6-31G(d) optimized geometries through the IEFPCM model (in MeOH). The overall calculated ECD curve was generated using SpecDis 1.604 [3] with σ = 0.30 eV.

Table S1 Standard orientation of (*R*)-**1a-**a at B3LYP/6-31G(d) level in gas phase

Center Number	Atomic Number	Atomic Type	Coordinates (Angstroms) X	Y	Z
1	6	0	-2.082089	-2.076633	0.115284
2	6	0	-2.708479	0.805360	-0.782131
3	6	0	-1.168174	-0.889691	0.305609
4	6	0	-1.437567	0.349731	-0.097050
5	6	0	0.108960	-1.238462	1.054014
6	6	0	-0.517015	1.506068	0.191871
7	6	0	1.389886	-1.043972	0.247703
8	6	0	0.966968	1.440230	0.055216
9	6	0	1.793342	0.402491	0.032562
10	6	0	3.266446	0.594838	-0.224217
11	8	0	-0.999987	2.579084	0.426246
12	8	0	1.357202	-1.734331	-0.978130
13	1	0	-2.982578	-1.857595	-0.436661
14	1	0	-1.547026	-2.868505	-0.402318
15	1	0	-2.372376	-2.475334	1.084272
16	1	0	-3.324338	-0.017643	-1.111005
17	1	0	-3.290048	1.426410	-0.111511
18	1	0	-2.473942	1.414930	-1.648546
19	1	0	0.182567	-0.668595	1.975611
20	1	0	0.066960	-2.282954	1.339332
21	1	0	2.190587	-1.516851	0.803484
22	1	0	1.374886	2.428794	-0.066076
23	1	0	3.846895	0.318614	0.652518
24	1	0	3.585929	-0.050522	-1.036506
25	1	0	3.501779	1.619881	-0.480496
26	1	0	0.709324	-1.328414	-1.540187

Table S2 Standard orientation of (*R*)-**1a-**b at B3LYP/6-31G(d) level in gas phase

Center Number	Atomic Number	Atomic Type	Coordinates (Angstroms) X	Y	Z
1	6	0	-1.894275	-2.205820	-0.006892
2	6	0	-2.757224	0.650236	-0.831329
3	6	0	-1.119445	-0.942358	0.273756
4	6	0	-1.485669	0.276563	-0.103224
5	6	0	0.143518	-1.174275	1.085584
6	6	0	-0.645898	1.483346	0.214790
7	6	0	1.452413	-0.918854	0.344944
8	6	0	0.833246	1.507096	0.013676
9	6	0	1.725063	0.526541	-0.009746
10	6	0	3.164347	0.805189	-0.364605
11	8	0	-1.182981	2.515889	0.505388
12	8	0	1.552794	-1.669621	-0.842759
13	1	0	-2.779493	-2.053604	-0.604237
14	1	0	-1.253966	-2.909292	-0.533102
15	1	0	-2.193584	-2.681069	0.924385
16	1	0	-3.379862	-0.202688	-1.054335
17	1	0	-3.329408	1.347342	-0.231949
18	1	0	-2.526849	1.150884	-1.767367
19	1	0	0.138338	-0.569031	1.987382
20	1	0	0.156390	-2.206905	1.423374
21	1	0	2.254837	-1.214617	1.020623
22	1	0	1.171137	2.512380	-0.169813
23	1	0	3.821759	0.577303	0.470968
24	1	0	3.465895	0.176020	-1.195280
25	1	0	3.313831	1.840478	-0.642769
26	1	0	1.686020	-2.583327	-0.630652

Table S3 Standard orientation of (*R*)-**7a -**a at B3LYP/6-31G(d) level in gas phase

Center Number	Atomic Number	Atomic Type	Coordinates (Angstroms) X	Y	Z
1	6	0	0.164739	0.406967	0.415655
2	6	0	1.322253	-0.373322	0.326856
3	6	0	1.319253	-1.702185	-0.094455
4	6	0	0.114365	-2.272304	-0.479771
5	6	0	-1.074276	-1.521362	-0.444649
6	6	0	-1.014298	-0.195550	0.022512
7	6	0	-2.338055	-2.077791	-0.911488
8	6	0	-3.504853	-1.119856	-0.950505
9	6	0	-3.384189	-0.076992	0.138313
10	8	0	-2.131994	0.561119	0.080721
11	6	0	0.213972	1.845626	0.897043
12	6	0	0.514463	2.818155	-0.219788
13	6	0	-0.220267	3.827777	-0.661854
14	6	0	0.268702	4.698650	-1.795841
15	6	0	-1.572719	4.229773	-0.124804
16	8	0	2.460400	0.201705	0.720109

17	6	0	3.757275	-0.311557	0.365321
18	6	0	3.714962	-1.808265	0.181515
19	6	0	2.582224	-2.441411	-0.058773
20	6	0	4.659446	0.078746	1.530933
21	6	0	4.192600	0.383352	-0.926172
22	8	0	-2.463810	-3.214758	-1.301903
23	8	0	0.129206	-3.536153	-0.884298
24	8	0	-3.580332	-0.705361	1.344683
25	6	0	-3.627123	0.125652	2.474506
26	1	0	-4.427640	-1.673431	-0.851858
27	1	0	-3.494434	-0.630270	-1.920636
28	1	0	-4.093985	0.729785	0.005819
29	1	0	0.993492	1.928513	1.645701
30	1	0	-0.717634	2.086267	1.384916
31	1	0	1.463170	2.643338	-0.702365
32	1	0	0.337639	5.738855	-1.484493
33	1	0	1.244276	4.388998	-2.151639
34	1	0	-0.422665	4.667007	-2.635245
35	1	0	-2.304183	4.250181	-0.929423
36	1	0	-1.944506	3.565061	0.641187
37	1	0	-1.533204	5.235265	0.288742
38	1	0	4.654728	-2.329936	0.223223
39	1	0	2.552914	-3.502260	-0.217102
40	1	0	4.325657	-0.402337	2.442516
41	1	0	5.682708	-0.223393	1.333600
42	1	0	4.639914	1.152532	1.678450
43	1	0	5.180318	0.045037	-1.222260
44	1	0	3.502716	0.154302	-1.730408
45	1	0	4.222665	1.458914	-0.786551
46	1	0	-0.758266	-3.807374	-1.125181
47	1	0	-3.912144	-0.498230	3.308446
48	1	0	-2.662836	0.574879	2.675731
49	1	0	-4.367290	0.911266	2.347024

Table S4 Standard orientation of (*R*)-**7a** -b at B3LYP/6-31G(d) level in gas phase

Center	Atomic	Atomic	Coordinates (Angstroms)		
Number	Number	Type	X	Y	Z
1	6	0	0.172158	0.290310	-0.721283
2	6	0	1.416537	-0.255404	-0.389069
3	6	0	1.586643	-1.569533	0.043442
4	6	0	0.466195	-2.380831	0.151488
5	6	0	-0.810221	-1.881898	-0.164890
6	6	0	-0.921622	-0.541917	-0.580044
7	6	0	-1.989896	-2.737618	-0.126631
8	6	0	-3.269629	-2.117719	-0.635035
9	6	0	-3.286690	-0.628586	-0.371219
10	8	0	-2.127875	-0.019540	-0.889874
11	6	0	0.045562	1.719103	-1.217450
12	6	0	-0.040690	2.730955	-0.097981

13	6	0	-0.901385	3.728113	0.051771
14	6	0	-0.797170	4.667675	1.231094
15	6	0	-2.030790	4.059162	-0.894585
16	8	0	2.475248	0.543184	-0.537451
17	6	0	3.742558	0.286124	0.094583
18	6	0	3.958016	-1.193522	0.293710
19	6	0	2.948404	-2.043503	0.295568
20	6	0	3.748000	1.022153	1.435723
21	6	0	4.776656	0.873874	-0.859419
22	8	0	-1.965788	-3.892913	0.228038
23	8	0	0.646545	-3.635035	0.545523
24	8	0	-3.396059	-0.447191	0.985061
25	6	0	-3.554265	0.878899	1.422163
26	1	0	-4.114187	-2.601050	-0.165144
27	1	0	-3.317727	-2.296298	-1.705943
28	1	0	-4.095764	-0.133414	-0.893292
29	1	0	-0.808150	1.786288	-1.873363
30	1	0	0.920931	1.944887	-1.816712
31	1	0	0.727443	2.620133	0.650763
32	1	0	-1.711772	4.657018	1.820349
33	1	0	0.027017	4.406563	1.884316
34	1	0	-0.651546	5.692920	0.897621
35	1	0	-2.971615	4.125195	-0.352810
36	1	0	-1.866429	5.031646	-1.353541
37	1	0	-2.154212	3.334191	-1.686359
38	1	0	4.968883	-1.518432	0.465837
39	1	0	3.096552	-3.092809	0.463762
40	1	0	2.962806	0.641921	2.079318
41	1	0	4.697463	0.878456	1.941248
42	1	0	3.593529	2.085515	1.285247
43	1	0	5.773682	0.774056	-0.443153
44	1	0	4.745528	0.358505	-1.811909
45	1	0	4.577620	1.926075	-1.027970
46	1	0	-0.197517	-4.089298	0.569677
47	1	0	-3.731566	0.834533	2.486512
48	1	0	-2.668197	1.466851	1.225534
49	1	0	-4.409567	1.343482	0.938023

Table S5 Standard orientation of (*R*)-**7a-c** at B3LYP/6-31G(d) level in gas phase

Center	Atomic	Atomic	Coordinates (Angstroms)		
Number	Number	Type	X	Y	Z
1	6	0	0.063670	0.458521	0.323919
2	6	0	0.767715	-0.751063	0.327004
3	6	0	0.182456	-1.976538	0.012253
4	6	0	-1.154958	-1.997901	-0.358228
5	6	0	-1.898646	-0.806016	-0.415474
6	6	0	-1.262988	0.396036	-0.052984
7	6	0	-3.284105	-0.795763	-0.867967
8	6	0	-3.920874	0.566125	-1.012820

9	6	0	-3.342781	1.543969	-0.013918
10	8	0	-1.938294	1.566625	-0.096068
11	6	0	0.738851	1.765198	0.696464
12	6	0	1.353466	2.461084	-0.496023
13	6	0	2.593033	2.901985	-0.651182
14	6	0	3.009252	3.601333	-1.924394
15	6	0	3.697795	2.786795	0.371420
16	8	0	2.043891	-0.701257	0.710721
17	6	0	2.983501	-1.741815	0.386933
18	6	0	2.298924	-3.086032	0.344739
19	6	0	0.998620	-3.185189	0.141621
20	6	0	4.022659	-1.683693	1.500682
21	6	0	3.605711	-1.403567	-0.969021
22	8	0	-3.895795	-1.793904	-1.168903
23	8	0	-1.694763	-3.172167	-0.659225
24	8	0	-3.773461	1.171415	1.236354
25	6	0	-3.441004	2.038538	2.288882
26	1	0	-4.990629	0.477219	-0.888510
27	1	0	-3.716086	0.917505	-2.020604
28	1	0	-3.632852	2.563983	-0.232279
29	1	0	1.473441	1.573297	1.462961
30	1	0	-0.005982	2.423370	1.129837
31	1	0	0.661772	2.619328	-1.308809
32	1	0	3.821915	3.067668	-2.412623
33	1	0	2.189064	3.681905	-2.627939
34	1	0	3.373792	4.604727	-1.714530
35	1	0	4.577095	2.326548	-0.073406
36	1	0	4.001733	3.774108	0.712789
37	1	0	3.421047	2.203569	1.237759
38	1	0	2.921586	-3.956350	0.453516
39	1	0	0.507870	-4.137511	0.081585
40	1	0	3.561467	-1.897864	2.457467
41	1	0	4.806661	-2.412302	1.322643
42	1	0	4.472803	-0.698629	1.545155
43	1	0	4.346010	-2.149568	-1.239872
44	1	0	2.845384	-1.386699	-1.741264
45	1	0	4.085857	-0.431934	-0.933605
46	1	0	-2.614762	-3.051126	-0.900285
47	1	0	-3.952755	1.673008	3.166603
48	1	0	-2.374200	2.047243	2.472574
49	1	0	-3.774642	3.050759	2.076383

Reference

[1] Al-Jallal AN,El-Azhary AA. Conformational Study of the Structure of dibenzo-18-crown-6. Comparison with 18-crown-6 [J]. *J MOL GRAPH MODEL*, 75 (2017): 49-54.

[2] Ozcelikay G, Karadas-Bakirhan N, Taskin-Tok T, *et al*. A selective and molecular

imaging approach for anticancer drug: Pemetrexed by nanoparticle accelerated molecularly imprinting polymer [J]. *Electrochimica Acta*, 2020, 354:136665.

[3] Torsten B, Anu S, Yasmin H, *et al*. SpecDis: quantifying the comparison of calculated and experimental electronic circular dichroism spectra [J]. Chirality,2013, **25** (4): 243-249.

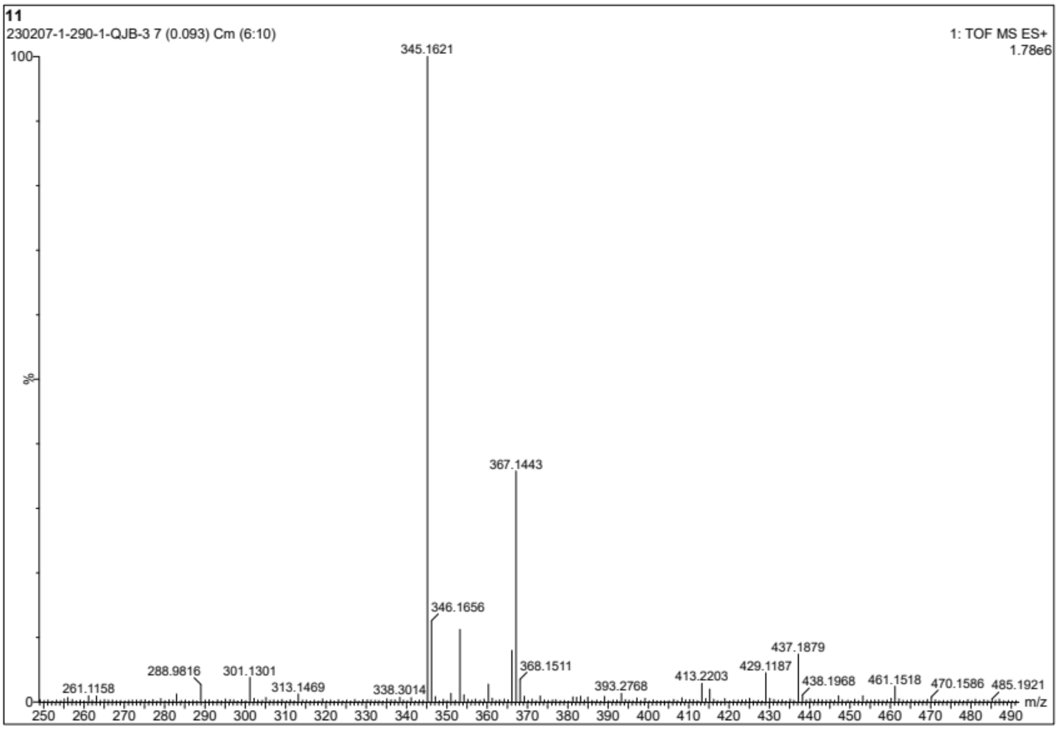

Fig. S1 (+) HR-ESI-MS sepectrum of compound **1**

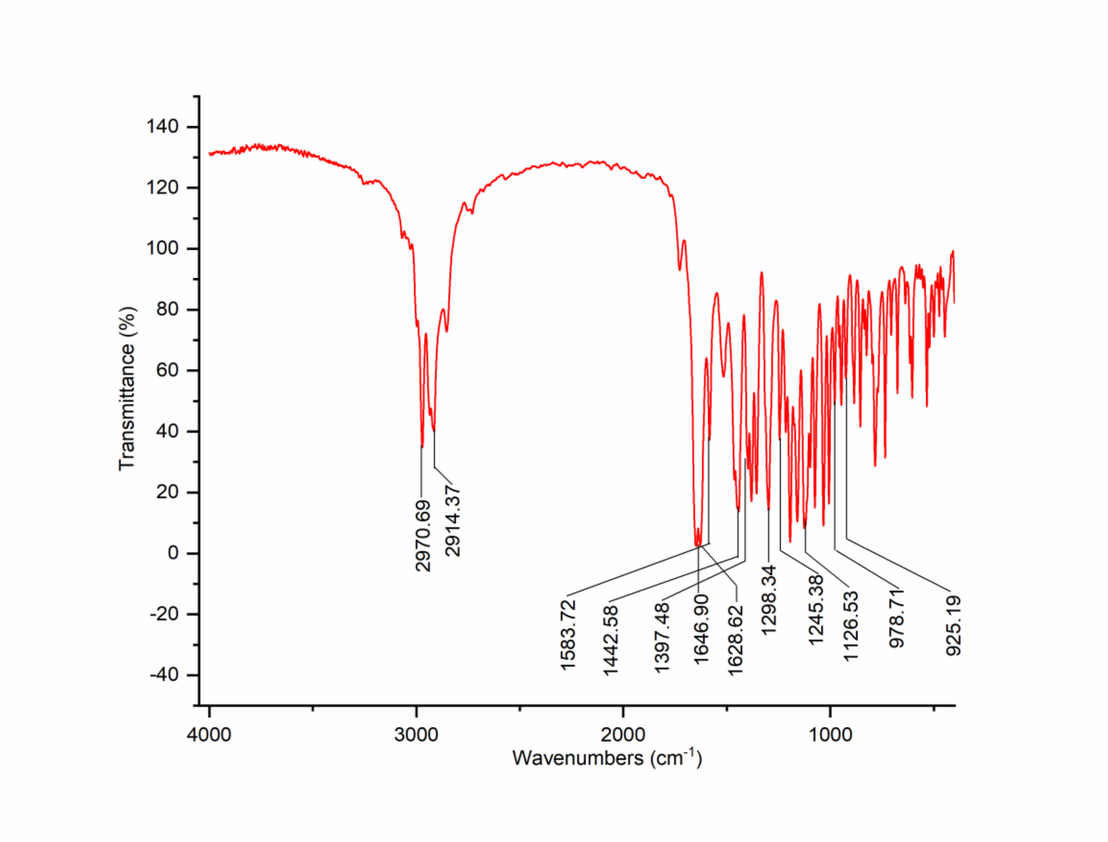

Fig. S2 IR spectrum of compound **1**

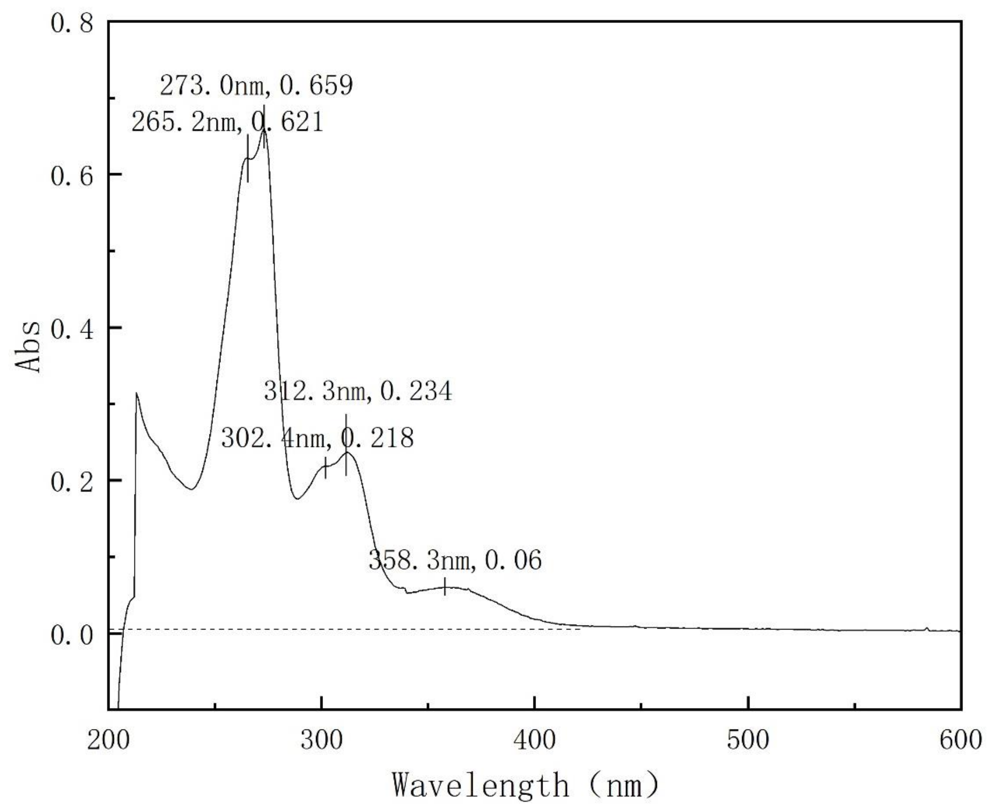

Fig. S3 UV spectrum of compound **1** in MeOH

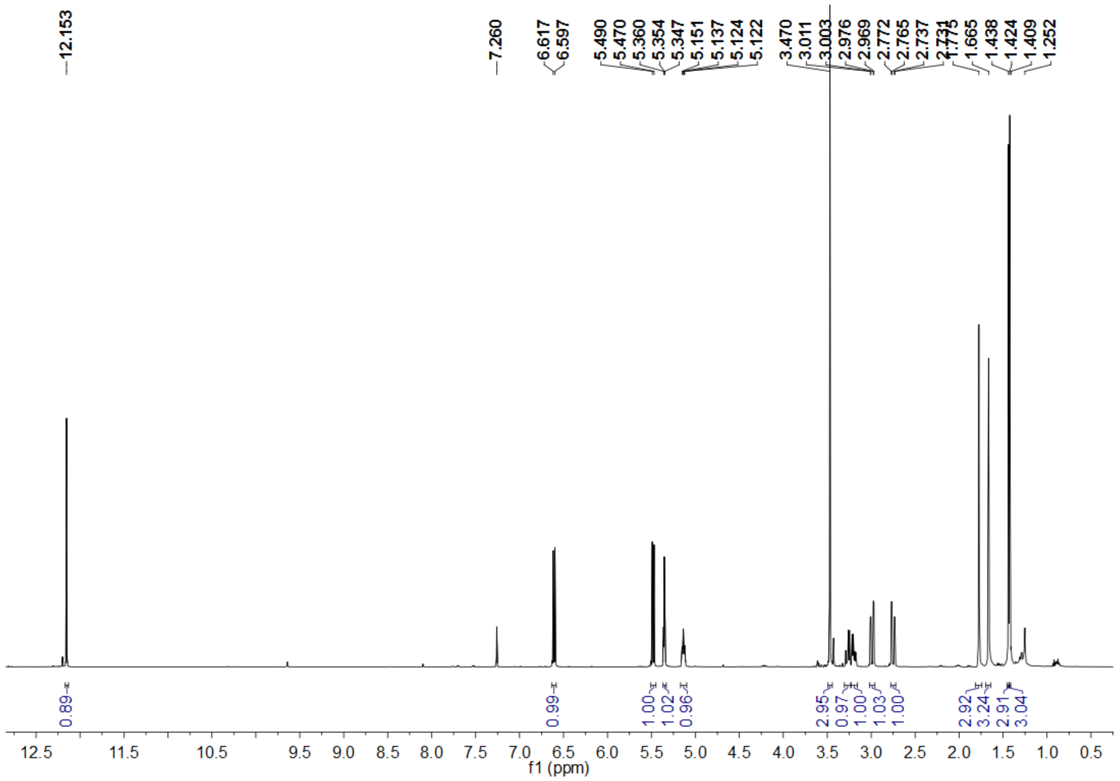

Fig. S4 ^{1}H NMR sepectrum of compound **1** in $CDCl_3$

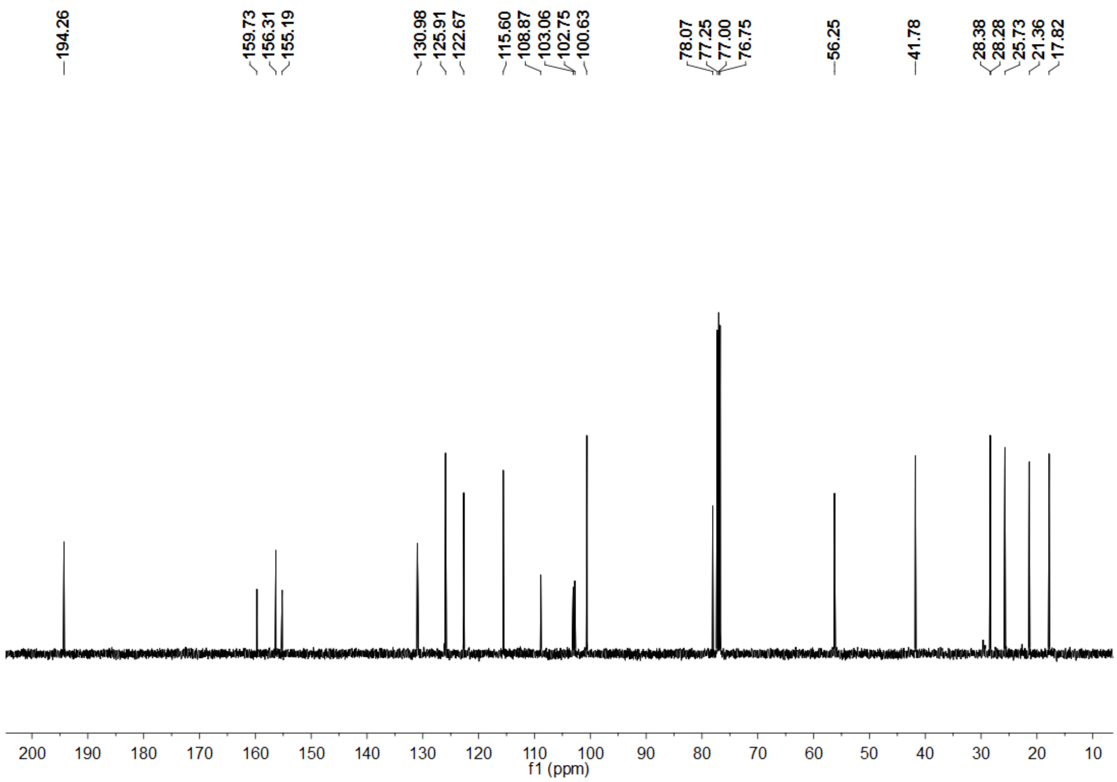

Fig. S5 ^{13}C NMR sepectrum of compound **1** in $CDCl_3$

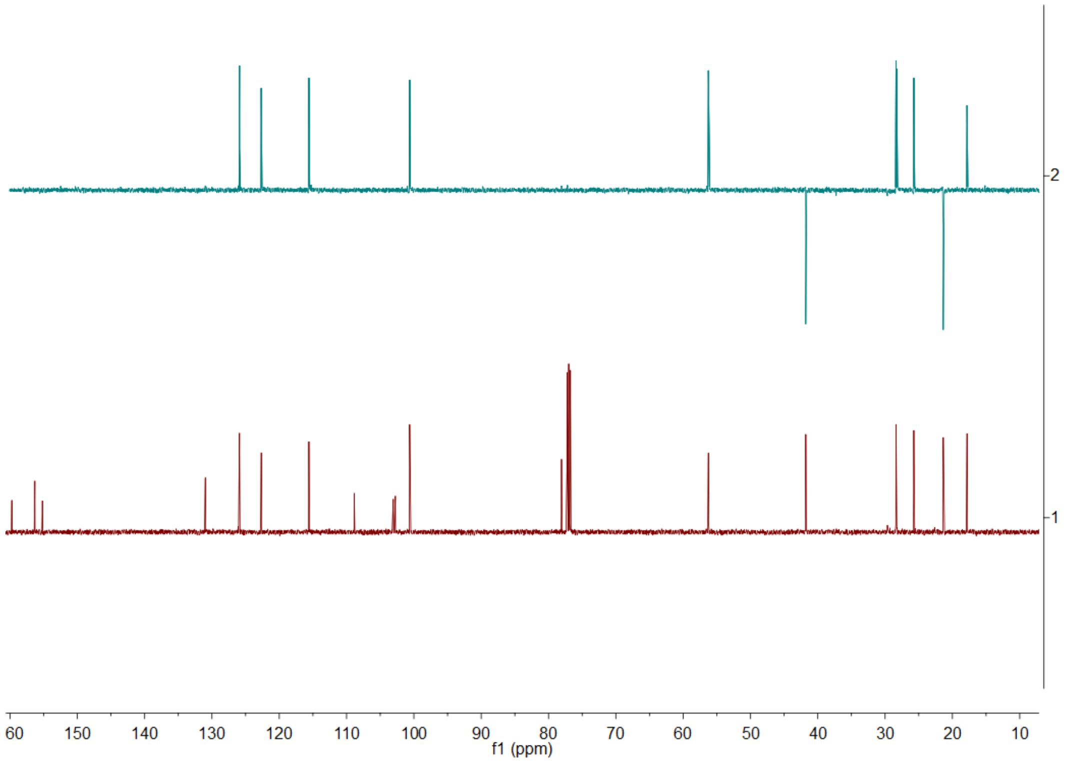

Fig. S6 DEPT-135 spectrum of compound **1** in $CDCl_3$

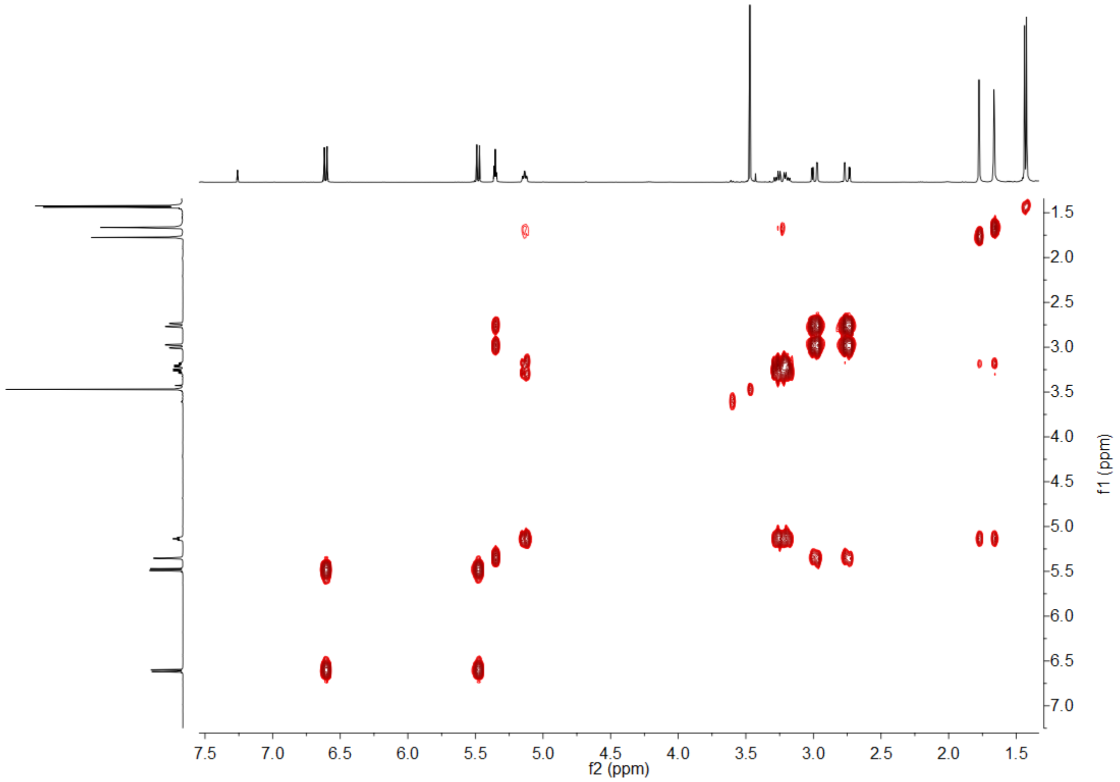

Fig. S7 ^{1}H-^{1}H COSY spectrum of compound **1** in $CDCl_3$

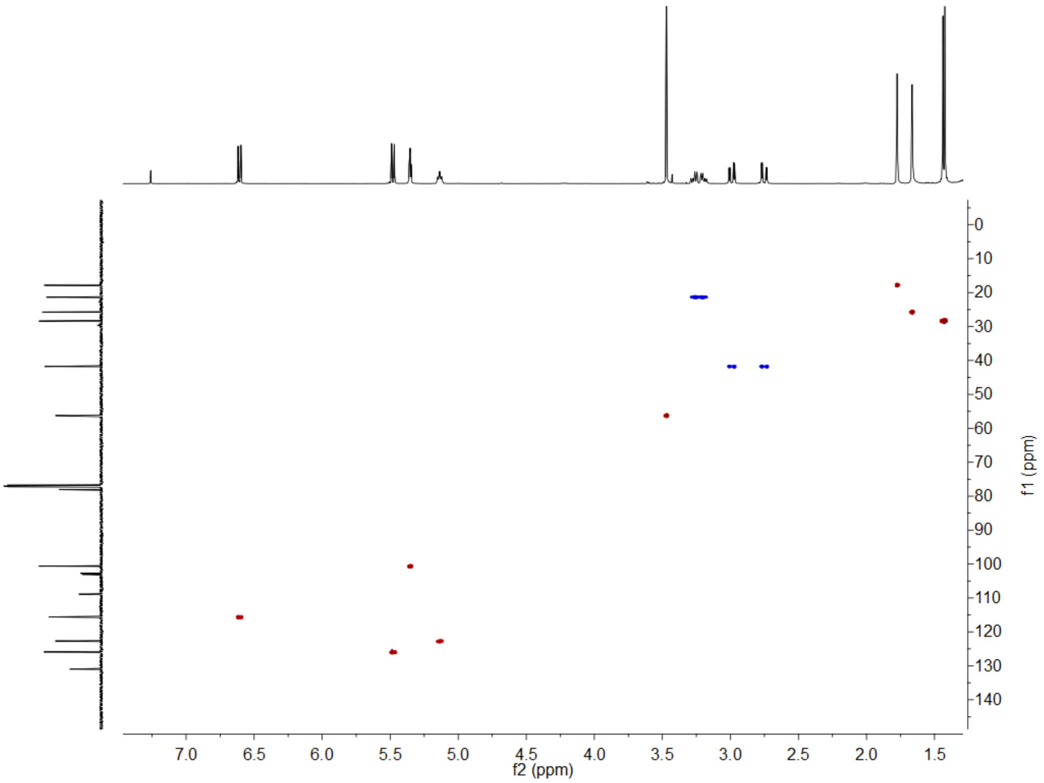

Fig. S8 HSQC spectrum of compound **1** in $CDCl_3$

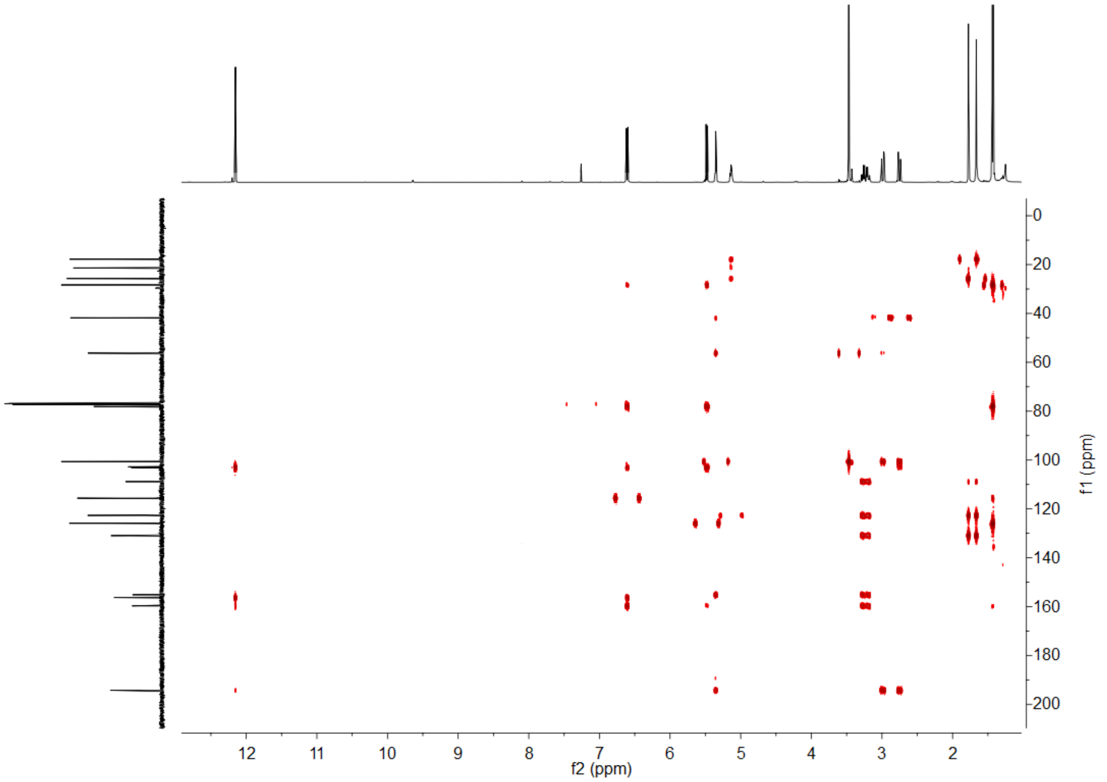

Fig. S9 HMBC spectrum of compound **1** in $CDCl_3$

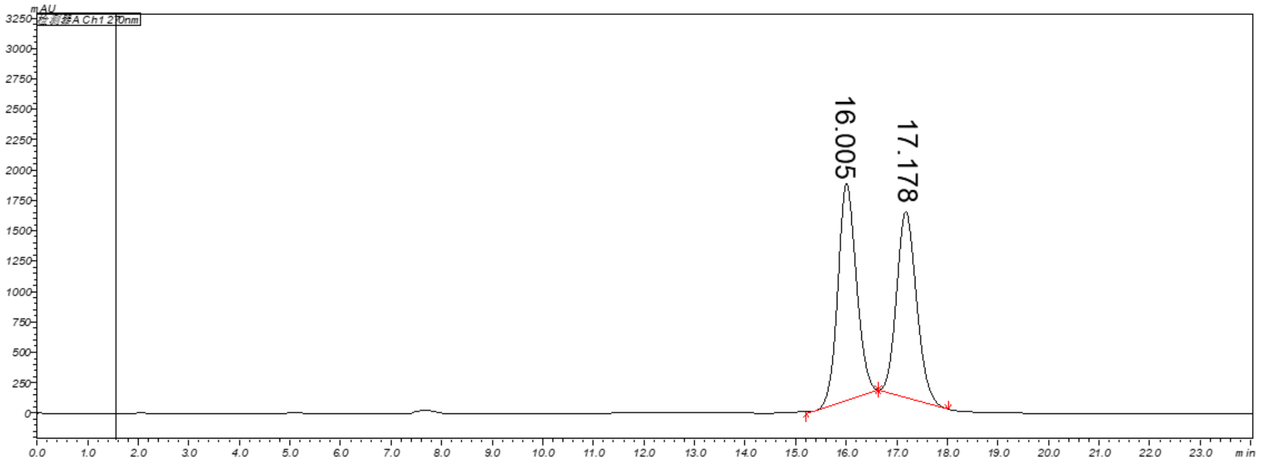

CHiRALONE 5-C column, *n*-hexane/isopropanol (90:10), 2 mL/min

Compound	Retention Time (min)	% Area
1a	16.005	50.500
1b	17.178	49.500

Fig. S10 Chiral HPLC analysis of compound **1**

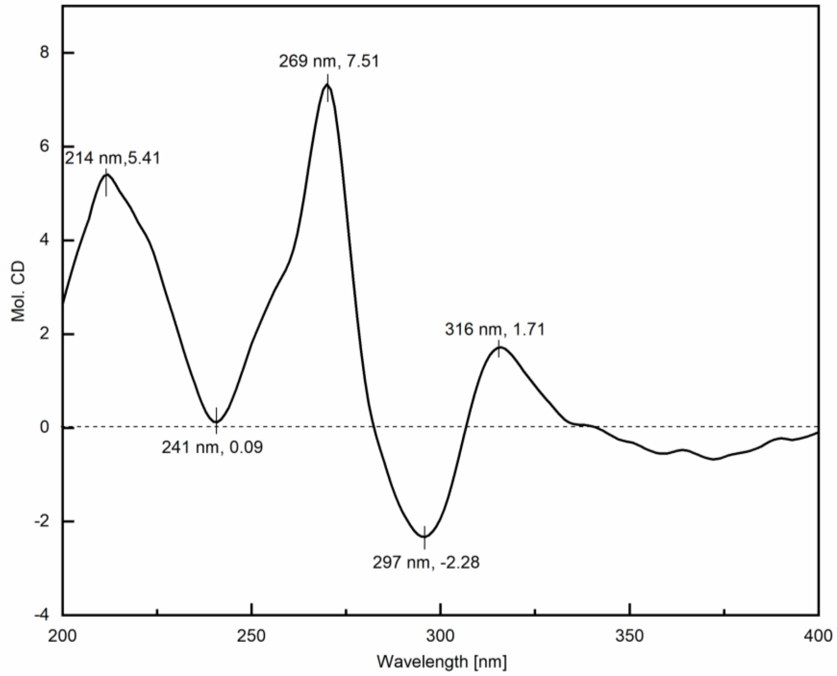

Fig. S11 ECD spectrum of compound **1a** in MeOH

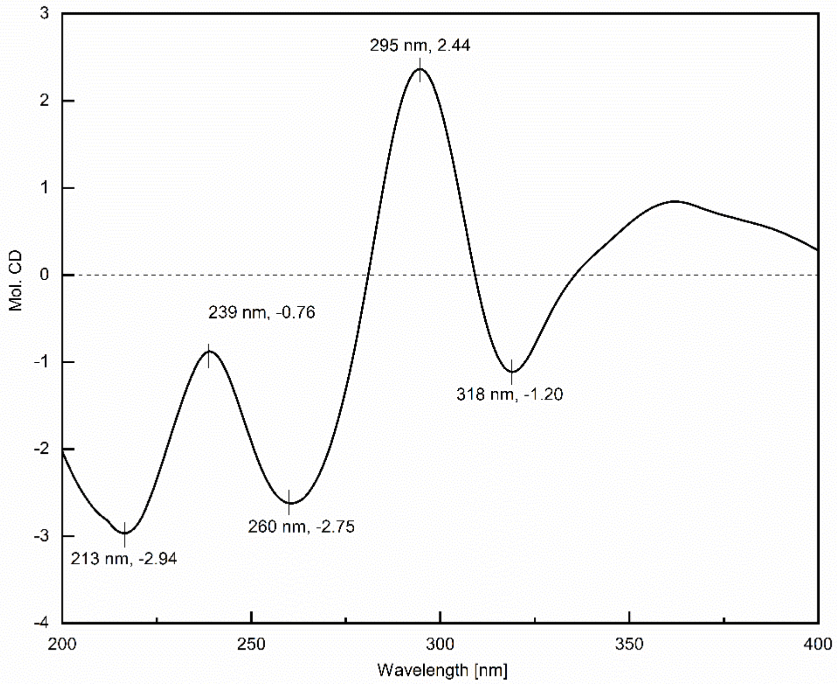

Fig. S12 ECD spectrum of compound **1b** in MeOH

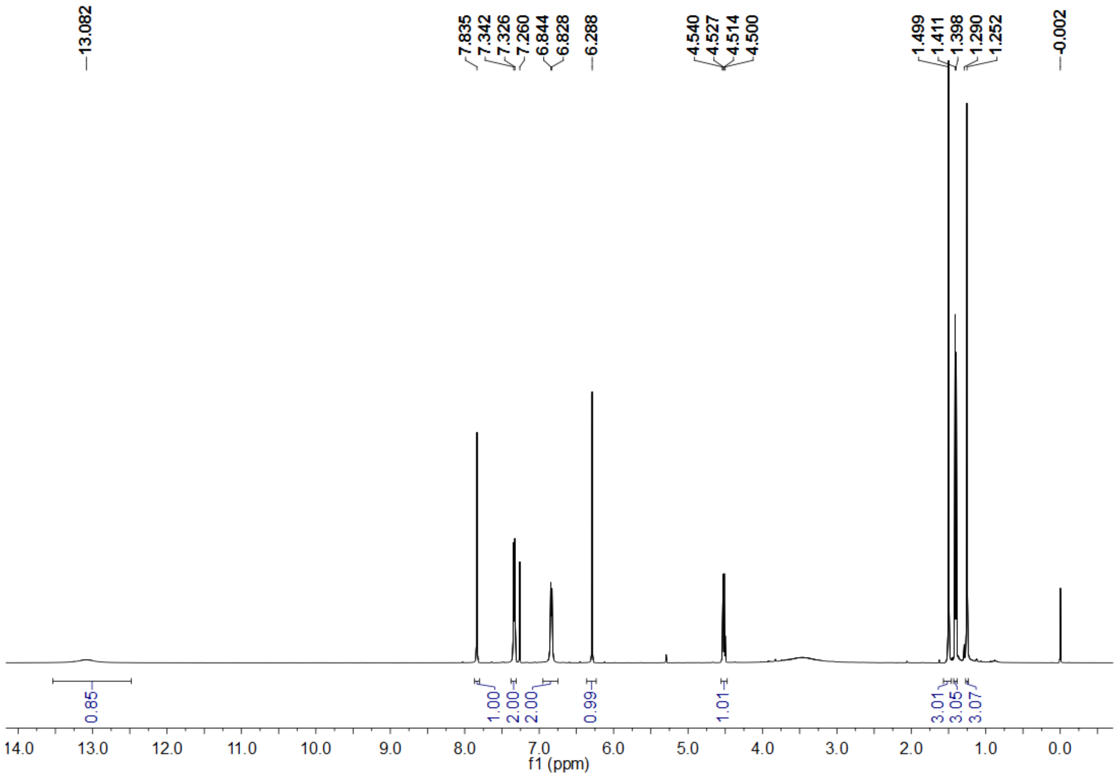

Fig. S13 ^{1}H NMR sepectrum of compound **7** in $CDCl_3$

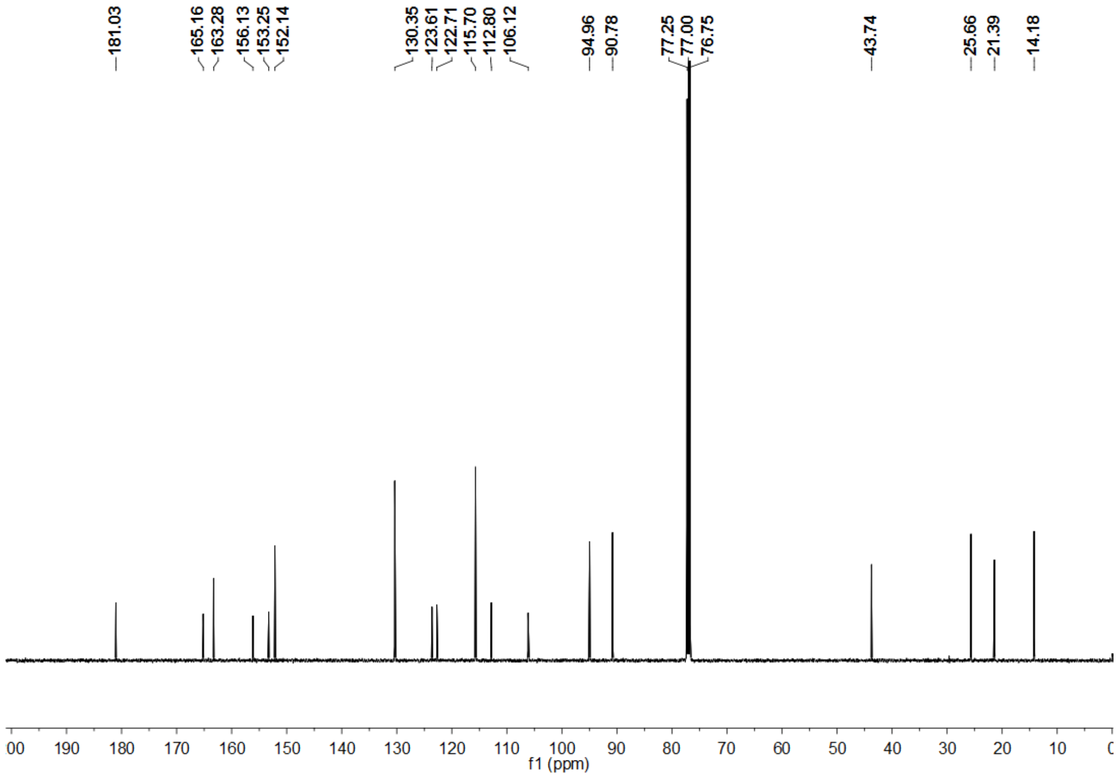

Fig. S14 ^{13}C NMR sepectrum of compound **7** in $CDCl_3$

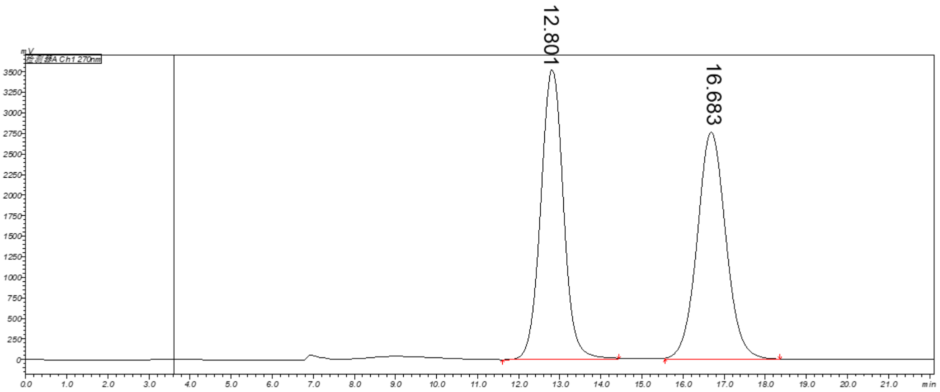

CHiRALONE 5-C column, *n*-hexane/isopropanol (90:10), 2 mL/min

Compound	Retention Time (min)	% Area
7a	12.801	51.58
7b	16.683	48.42

Fig. S15 Chiral HPLC analysis of compound **7**

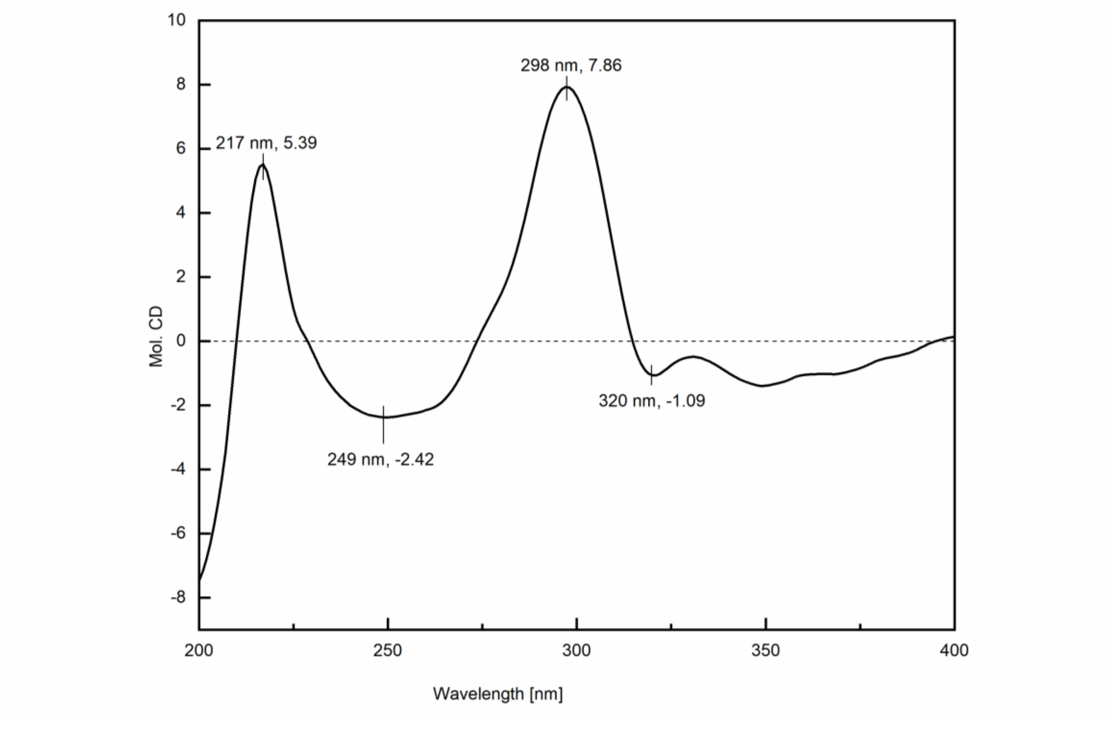

Fig. S16 ECD spectrum of compound **7a** in MeOH

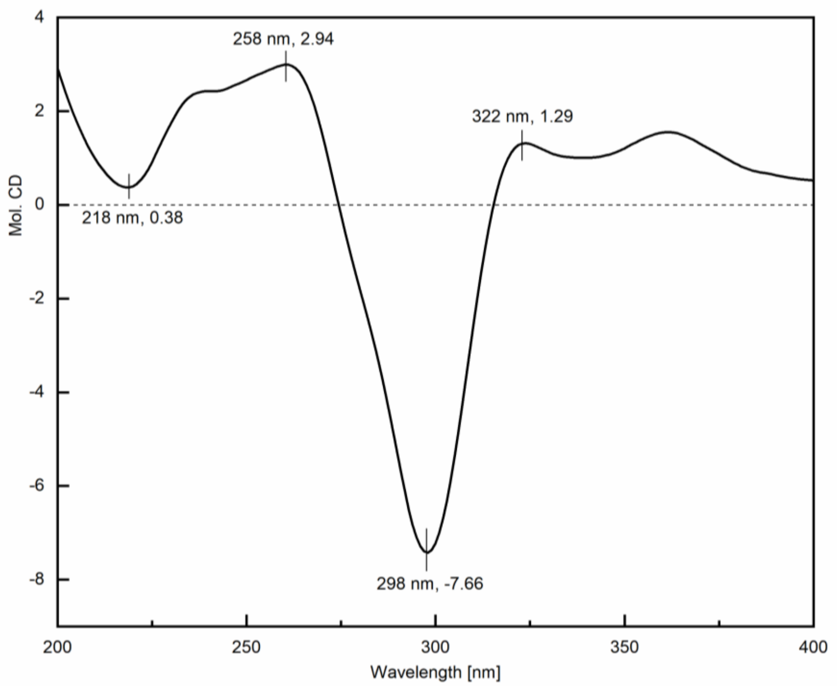

Fig. S17 ECD spectrum of compound **7b** in MeOH

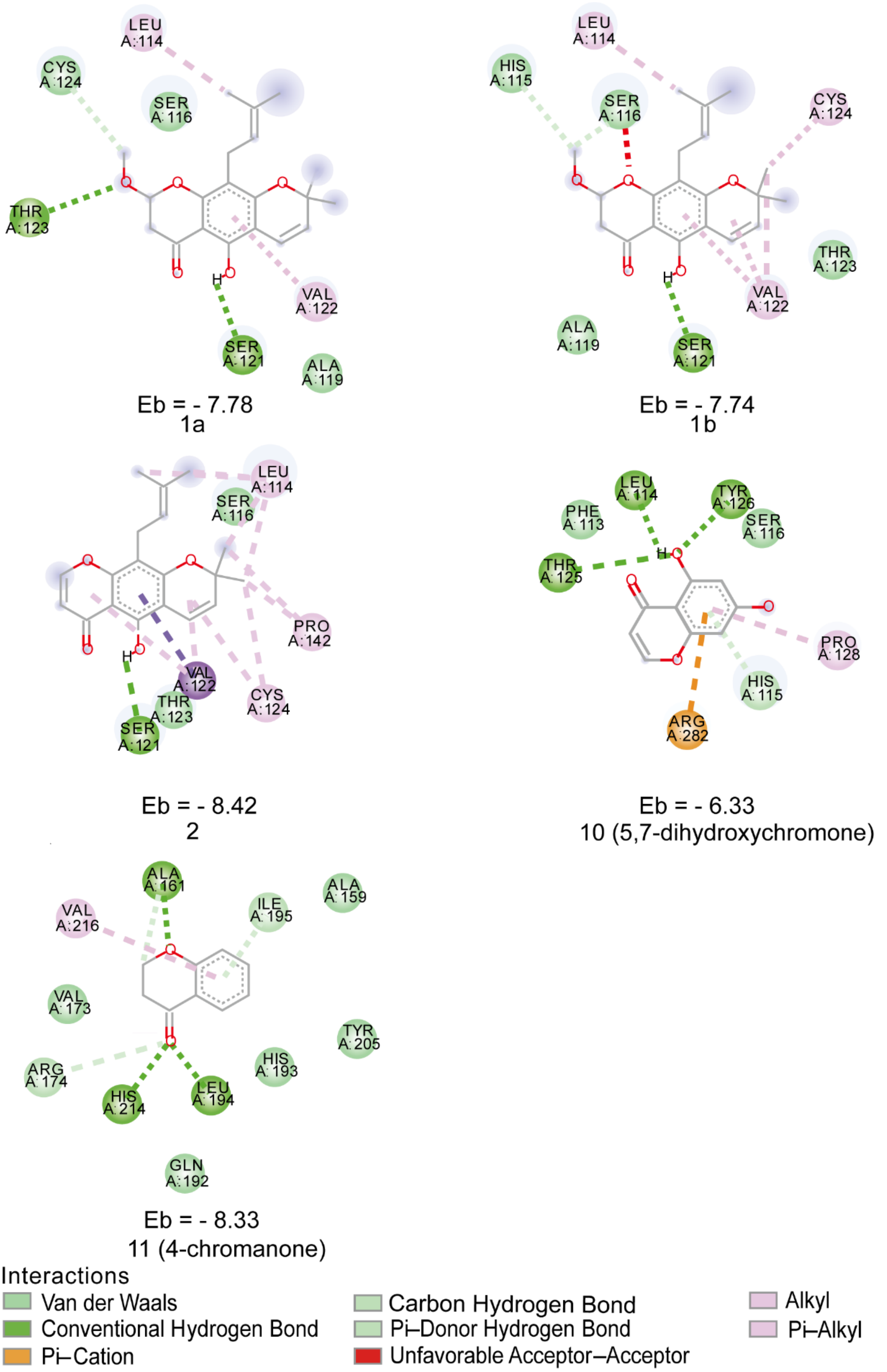

Fig. S18 The molecule docking results of chromones and chromanones with p53

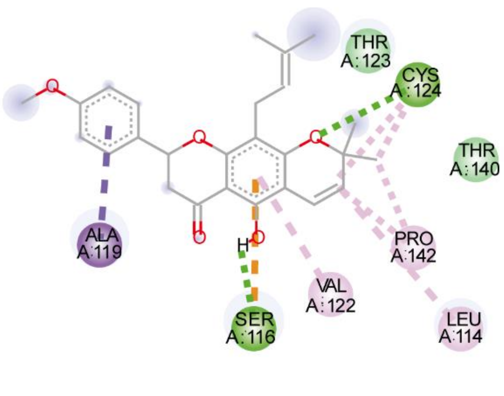

Eb = - 8.44
3

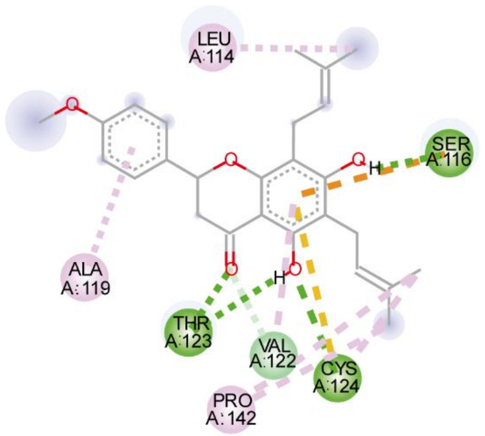

Eb = - 8.17
4

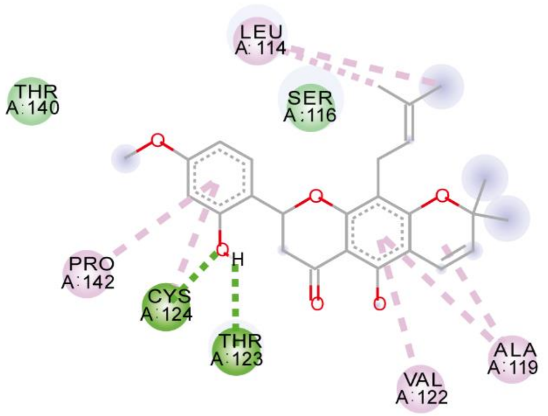

Eb = - 9.0
5

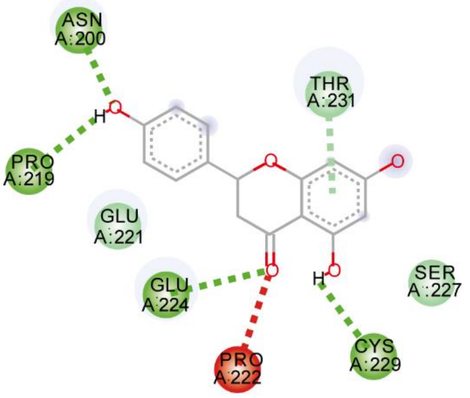

Eb = - 7.23
naringenin

Interactions
- Van der Waals
- Conventional Hydrogen Bond
- Pi–Cation
- Carbon Hydrogen Bond
- Unfavorable Acceptor–Acceptor
- Pi–Donor Hydrogen Bond
- Pi–Sulfur
- Pi–Sigma
- Alkyl
- Pi–Alkyl

Fig. S19 The molecule docking results of flavanones with p53

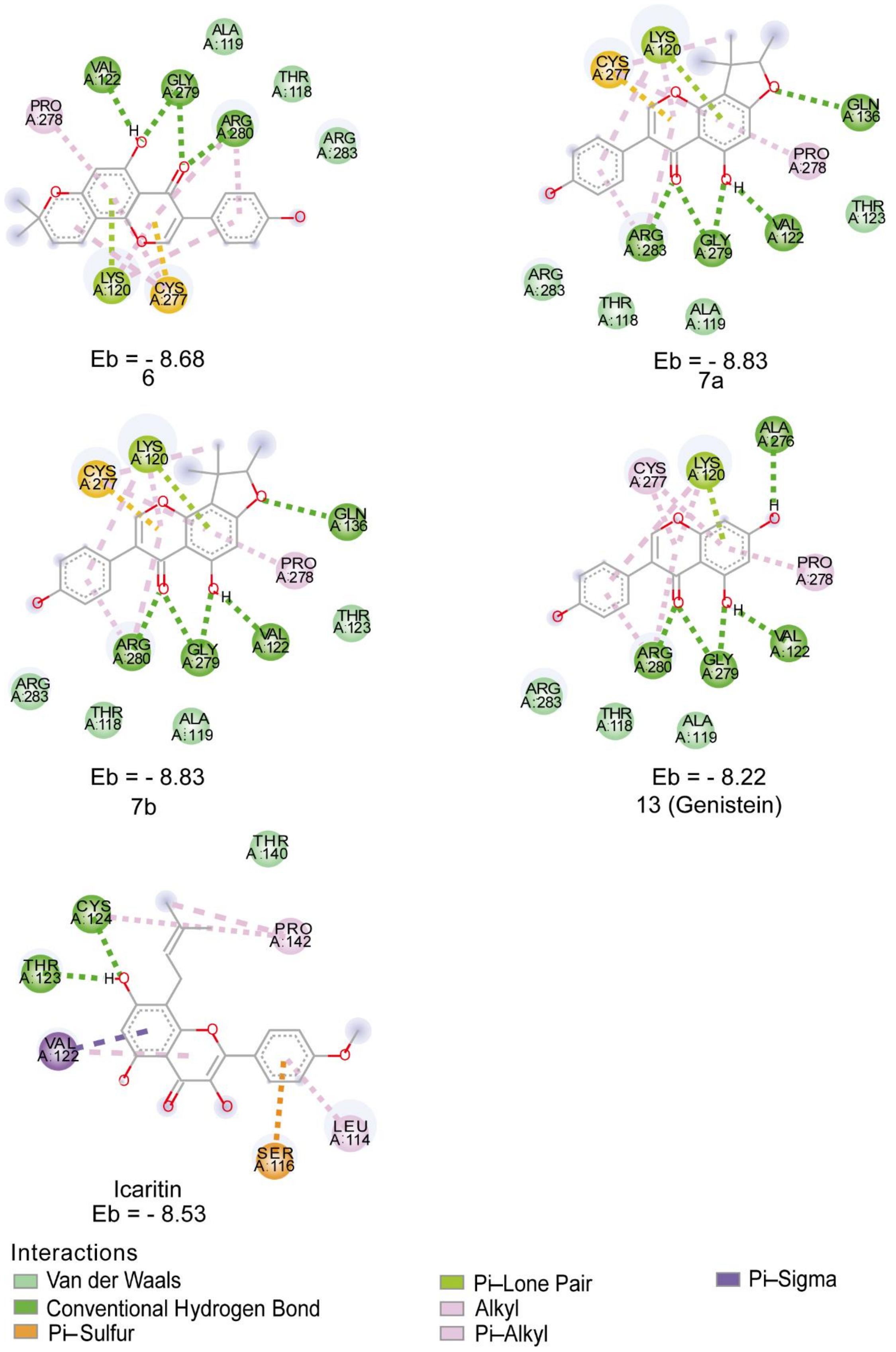

Fig. S20 The molecule docking results of isoflavones and icaritin with p53
